# Transgenic and knockout analyses of *Masculinizer* and *doublesex* illuminated the unique functions of *doublesex* in germ cell sexual development of the silkworm, *Bombyx mori*

**DOI:** 10.1186/s12861-020-00224-2

**Published:** 2020-09-21

**Authors:** Tomohisa Yuzawa, Misato Matsuoka, Megumi Sumitani, Fugaku Aoki, Hideki Sezutsu, Masataka G. Suzuki

**Affiliations:** 1 AIR WATER INC, 4-9-4 Hatchobori, Chuo-ku, Tokyo, 104-0032 Japan; 2 Department of Integrated Biosciences, Graduate School of Frontier Sciences, The University of Tokyo, 5-1-5 Kashiwanoha, Kashiwa-shi, Chiba, 277-8562 Japan; 3 SHINYUSHA, 1-12 Kanda Jimbocho, Chiyoda-ku, Tokyo, 101-0051 Japan; 4 Genetically Modified Organism Research Center, National Institute of Agrobiological Sciences, Owashi, Tsukuba, 305-8634 Japan

**Keywords:** *Bombyx mori*, Sex determination, Sexual differentiation, *Masc*, *Bmdsx*, Gonad, Germ cell, Alternative splicing

## Abstract

**Background:**

*Masculinizer* (*Masc*) plays a pivotal role in male sex determination in the silkworm, *Bombyx mori*. *Masc* is required for male-specific splicing of *B. mori doublesex* (*Bmdsx*) transcripts. The male isoform of *Bmdsx* (*BmdsxM*) induces male differentiation in somatic cells, while females express the female isoform of *Bmdsx* (*BmdsxF*), which promotes female differentiation in somatic cells. Our previous findings suggest that *Masc* could direct the differentiation of genetically female (ZW) germ cells into sperms. However, it remains unclear whether *Masc* directly induces spermatogenesis or if it promotes male differentiation in germ cells indirectly by inducing the expression of *BmdsxM*.

**Results:**

In this study, we performed genetic analyses using the transgenic line that expressed *Masc*, as well as various *Bmdsx* knockout lines. We found that *Masc*-expressing females with a homozygous mutation in *BmdsxM* showed normal development in ovaries. The formation of testis-like tissues was abolished in these females. On the other hand, *Masc*-expressing females carrying a homozygous mutation in *BmdsxF* exhibited almost complete male-specific development in gonads and germ cells. These results suggest that *BmdsxM* has an ability to induce male development in germ cells as well as internal genital organs, while *BmdsxF* inhibits *BmdsxM* activity and represses male differentiation. To investigate whether MASC directly controls male-specific splicing of *Bmdsx* and identify RNAs that form complexes with MASC in testes, we performed RNA immunoprecipitation (RIP) using an anti-MASC antibody. We found that MASC formed a complex with *AS1* lncRNA, which is a testis-specific factor involved in the male-specific splicing of *Bmdsx* pre-mRNA.

**Conclusions:**

Taken together, our findings suggest that *Masc* induces male differentiation in germ cells by enhancing the production of *BmdsxM*. Physical interaction between MASC and *AS1* lncRNA may be important for the *BmdsxM* expression in the testis. Unlike in the *Drosophila dsx*, *BmdsxM* was able to induce spermatogenesis in genetically female (ZW) germ cells. To the best of our knowledge, this is the first report that the role of *dsx* in germ cell sexual development is different between insect species.

## Background

Sex determination and sexual differentiation are strictly controlled by the sex-determination cascade that is composed of various proteins encoded by sex-determining genes. Although in all animal species, the females produce eggs as gametes while the males produce spermatozoa, there is high diversity in sex-determining genes among animal species [1]. For example, in vertebrates, the Sex-determining region Y (SRY) functions as a determinant of male differentiation in mammals [2]. In rainbow trout, the *sex-determining region Y* (*SdY*) [3], which is homologous to the immune system gene *interferon regulatory factor 9* (*IRF9*), triggers masculinization. In insects, the upstream regulators for maleness also exhibit significant diversity among species. A male determining factor *Nix* (*Nix*) in the mosquito *Aedes aegypti* [4], a maleness gene *Yob* in the malaria mosquito *Anopheles gambiae* [5], and the *Musca domestica male determiner* (*Mdmd*) in the housefly [6] differ in origin, structure, and mechanism of action.

In the silkworm, the chromosomal sex determination mechanism is distinct from those of mammals, mosquitoes and flies, with the female (ZW) being the heterogametic sex and the male (ZZ) the homogametic sex [7]. Accumulating evidence indicates that a Z-linked gene *Masculinizer* (*Masc*) acts as a key determinant for maleness in the silkworm [8, 9]. In individuals without the W chromosome, *Masc* induces male-specific splicing of the pre-mRNA of the *Insulin-like growth factor II mRNA binding protein* (*Imp*) gene [10]. The resulting male isoform of Imp (ImpM) cooperates with *Bombyx mori* P-element somatic inhibitor (BmPSI) to promote male-specific splicing of *Bmdsx*, which produces the male isoform of BmDSX (BmDSXM) [11, 12]. It has been recently reported that a long noncoding RNA transcribed from the *Bmdsx-AS1* gene (designated as *AS1* lincRNA) and the RNA binding protein BxRBP3A are also crucial for the male-specific splicing of *Bmdsx* [13, 14]. On the other hand, in individuals with the W chromosome, PIWI-interacting RNA (piRNA) is produced from the W-linked feminizer gene called *Feminizer* (*Fem*) [8]. The *Fem* piRNA-PIWI protein complex targets *Masc* mRNA for degradation. Insufficient levels of *Masc* expression result in the female-specific splicing of the *Bmdsx* pre-mRNA and subsequent production of the female isoform of BmDSX (BmDSXF) [8]. Morphological analyses using transgenic silkworms that ectopically express either *BmdsxF* or *BmdsxM*, as well as knockout silkworms homozygous for mutations in *Bmdsx*, suggested that *BmdsxF* and *BmdsxM* enhance female and male differentiation in gonads and external genitalia, respectively [15–18]. There have been no reports on the importance of *Bmdsx* in sexual differentiation in germ cells.

We previously demonstrated that transgenic expression of *Fem*-piRNA-resistant *Masc* gene (*Masc-R*) in females caused degenerated ovaries with testis-like tissues. Notably, the testis-like tissues produced a considerable number of sperms [9]. These findings strongly suggest that *Masc* could direct the differentiation of genetically female (ZW) germ cells into sperms. However, it remains unclear whether *Masc* directly induces spermatogenesis or if it promotes male differentiation in germ cells indirectly by inducing the expression of *BmdsxM*. To answer this question, it is necessary to clarify the importance of *Bmdsx* in sexual differentiation of germ cells.

*Bmdsx* is a *Bombyx* ortholog of *doublesex* (*dsx*) [8]. In most insect species, pre-mRNA from the *dsx* gene undergoes sexual dimorphic alternative splicing to yield female- and male-specific isoforms (*dsxF* and *dsxM*), which promote female and male differentiation, respectively, in somatic cells [15–20]. In the fruit fly *Drosophila melanogaster*, *dsxF* is dispensable for female differentiation in germ cells, and factors that are expressed only in genetically female germ cells, such as a protein product of *ovarian tumor* (*otu*), as well as the germline-specific isoforms of *ovo* and *Sex-lethal*, are essential for oogenesis [21–23]. Hence female differentiation of germ cells occurs even if the sex of the surrounding somatic cells is male. On the other hand, *dsxM* expression in the surrounding somatic cells is required for the male differentiation of germ cells. The protein product of *dsxM* in the surrounding somatic cells stimulates the JAK/STAT signaling pathway in germ cells, which plays an essential role in the initiation of spermatogenesis [21–23]. Complete spermatogenesis is achieved only when the sex of germ cells is male (XY) because multiple genes crucial for spermatogenesis are located on the Y chromosome [21–23]. These findings suggest that the role of *dsx* in sexual differentiation in Drosophila differ between somatic cells and germ cells. However, it remains unclear whether this is also the case in other insect species.

In this study, we performed further analysis using the *Masc-R* strain in combination with several *Bmdsx* knockout lines to elucidate whether *Masc* directly induces spermatogenesis or if it promotes male differentiation in germ cells indirectly by inducing the expression of *BmdsxM*. To this end, we performed genetic analyses in *Masc*-*R* females homozygous for mutations in either *BmdsxM* or *BmdsxF*. Furthermore, we performed RNA immunoprecipitation (RIP) analysis using an anti-MASC antibody to identify RNAs that interact with the MASC protein. The present study provides several lines of evidence that *Bmdsx* regulates sexual differentiation in germ cells in response to *Masc* expression and that the MASC protein interacts with *AS1* lncRNA, which is known to be involved in the male-specific splicing of *Bmdsx* pre-mRNA.

## Results

### *BmdsxM* knockout in *Masc*-*R* females fully restores ovary development

**Fig. 1:**
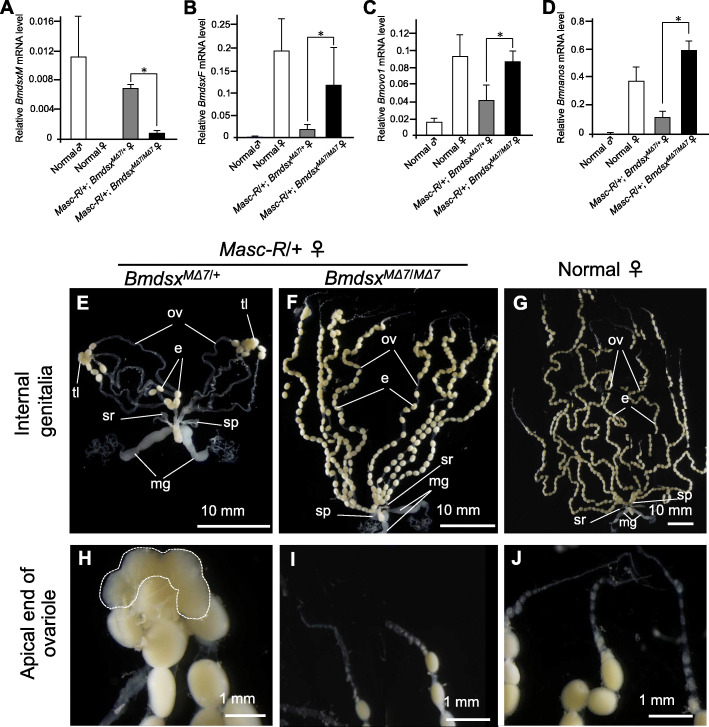
Morphological and molecular analysis of the internal genitalia of *Masc-R* females homozygous for *BmdsxM* mutation. Homozygous mutation in *BmdsxM* fully restored ovary development in *Masc-R* females. **a**, **b** The expression levels of *BmdsxM* (**a**) and *BmdsxF* (**b**) in animals with the indicated genotype were quantified by qRT-PCR. **c**, **d** Expression of *Bmovo-1* (**c**) and *Bm-nosO* (**d**), which is important for oogenesis, was quantified by qRT-PCR to evaluate the effect of *BmdsxM* knockout on gonadal sexual development in *Masc-R*/+ females. Error bars indicate standard deviation. * indicates a significant difference, as determined by Welch’s *t*-test. **e**–**g** Morphological analysis of the internal genitalia in *Masc-R* female heterozygous for *Bmdsx*^*MΔ7*^ (E), *Masc-R* female homozygous for *Bmdsx*^*MΔ7*^ (F), and normal female animals (**g**). e, egg; mg, mucous gland; ov, oviduct; sp., spermatheca; sr, seminal receptacle; tl, testis-like tissue. **h–j** High magnification images showing the apical end of the ovariole in *Masc-R* female heterozygous for *Bmdsx*^*MΔ7*^ **h**, *Masc-R* female homozygous for *Bmdsx*^*MΔ7*^(I), and normal female animals **j**. The dotted line indicates the testis-like tissues

Our previous study using the *Masc-R* strain suggested a potential role of *Masc* and *Bmdsx* in the sexual differentiation of the gonads and germ cells [9]. However, it remains unclear whether *Masc* directly induces spermatogenesis or promotes male differentiation in germ cells indirectly by inducing the expression of *BmdsxM*; the latter is supported by the fact that the ectopic expression of the *Masc* mRNA in *Masc-R* females results in the expression of both *BmdsxM* and *BmdsxF*. Therefore, to eliminate the influence of *BmdsxM* expression, we generated *Masc-R* females that do not express *BmdsxM* by crossing the *Masc-R* strain with a *BmdsxM* deletion mutant strain established in this study (Additional files 5 and 6). The *BmdsxM* mutant strain had a 7-bp deletion in the *Bmdsx* exon 5 that encodes for the male-specific open reading frame (ORF), producing a truncated version of the BmDSXM protein (Additional file 5). RT-PCR analysis using primers that can amplify both *BmdsxF* and *BmdsxM* at the same time revealed that transgenic expression of *Masc-R* reproducibly induced *BmdsxM* expression in females irrespective of the *Bmdsx* genotype (Additional file 8). qRT-PCR analysis demonstrated that the homozygous mutation in *BmdsxM* abolished *BmdsxM* expression in the *Masc-R* females, while it increased *BmdsxF* expression by as much as seven-fold compared with *Masc-R* females heterozygous for the *BmdsxM* mutation (Fig. 1a and b). Morphological analysis of the internal genitalia confirmed that *Masc-R* females heterozygous for the *BmdsxM* mutation (genetically the same as the *Masc-R*/+ females) formed degenerated ovaries (Fig. 1e), consistent with what has been previously reported for *Masc-R* females [9]. Testis-like tissues were observed at the apical end of ovarioles (Fig. 1h).

In comparison, *Masc-R* females homozygous for the *BmdsxM* mutation (thus only expressing *BmdsxF* even in the presence of the *Masc-R* gene) had fully developed ovaries as observed in normal females (Fig. 1f and g). Additionally, the morphological features of the apical end of ovarioles were similar to those observed in normal females (Fig. 1i and j). No testis-like tissues were observed in *Masc-R*/+, *Bmdsx*^*MΔ7*/ *MΔ7*^ females. These results demonstrated that the *Masc-R* transgene indirectly promotes the development of male gonads and the germ cells by inducing expression of *BmdsxM*.

### *BmdsxM* knockout in *Masc*-*R* females restores the ability of egg production

**Fig. 2:**
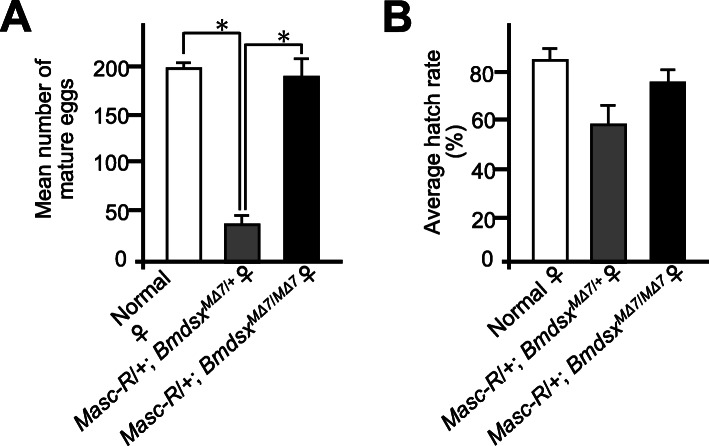
Fertility of *Masc-R* females homozygous for *BmdsxM* mutation. Homozygous mutation in *BmdsxM* fully restored the fertility of *Masc-R* females. **a** The mean number of mature eggs observed in adult ovaries. **b** Hatchability of eggs laid by females with the indicated genotype. Error bars indicate standard deviation. * indicates significant differences, as determined by Welch’s *t*-test

We next investigated the fertility of *Masc-R* females homozygous for the *BmdsxM* mutation. As reported previously, *Masc-R* expression in females caused a significant decrease in the number of mature eggs (Fig. 2a, *Masc-R*/+, *Bmdsx*^*MΔ7*/+^) [9]. Interestingly, the homozygous mutation in *BmdsxM* restored the number of mature eggs produced by *Masc-R* females (Fig. 2a, *Masc-R*/+, *Bmdsx*^*MΔ7*/ *MΔ7*^). The hatchability of eggs laid by the *Masc-R* females homozygous for the *BmdsxM* mutation was also similar to that of normal females (Fig. 2b). These results demonstrated that *BmdsxM* knockout fully restored the ability of egg production in *Masc-R* females.

### *BmdsxM* knockout in *Masc*-*R* females induces the expression of genes essential for oogenesis

**Fig. 3:**
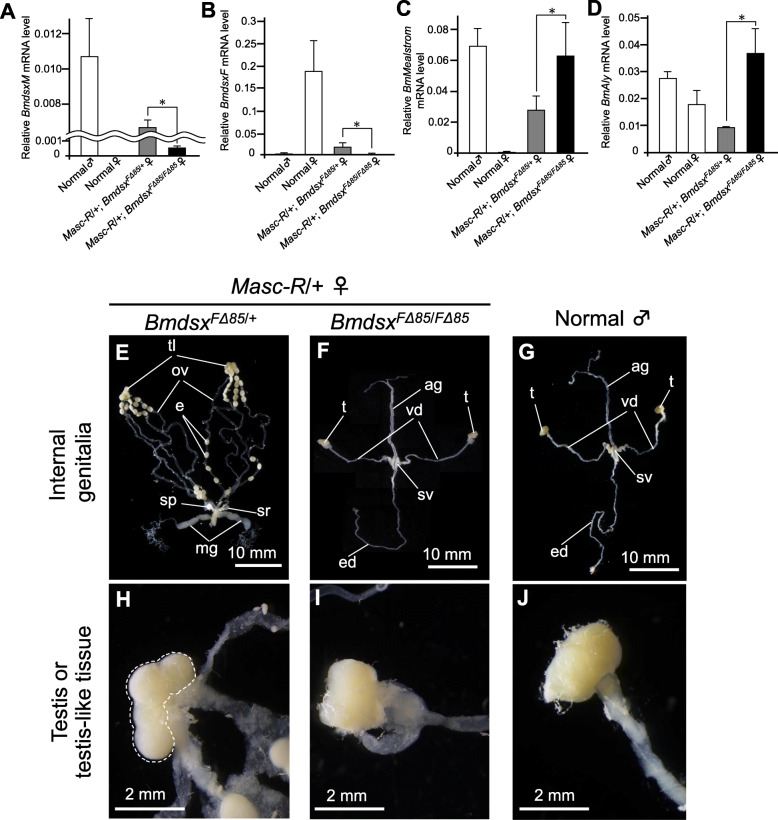
Morphological and molecular analysis of the internal genitalia of *Masc-R* females homozygous for *BmdsxF* mutation. Homozygous mutation in *BmdsxF* promoted female to male sex reversal in the internal genitalia of *Masc-R* females. (A, B) Expression levels of *BmdsxM* (**a**) and *BmdsxF* (**b**) in animals with the indicated genotype were quantified by qRT-PCR. Expression levels of *BmMaelstrom* (**c**) and *BmAly* (**d**), both of which are involved in spermatogenesis, were also quantified by qRT-PCR to evaluate the effect of *BmdsxF* knockout on gonadal and germ cell sexual development in *Masc-R*/+ females. Error bars indicate standard deviation. * indicates a significant difference, as determined by Welch’s *t*-test. **e–g** Morphological analysis of the internal genitalia in *Masc-R* female heterozygous for *Bmdsx*^*FΔ85*^ (**e**), *Masc-R* female homozygous for *Bmdsx*^*FΔ85*^ (**f**), and normal male animals (**g**). e, egg; mg, mucous gland; ov, oviduct; sp., spermatheca; sr, seminal receptacle; tl, testis-like tissue; ag, accessory gland; ed., ejaculatory duct; sv, seminal vesicle; t, testis; vd, vas deferens. **h–j** High magnification images showing testis-like tissues and testes in *Masc-R* female heterozygous for *Bmdsx*^*FΔ85*^ (**h**)*, Masc-R* female homozygous for *Bmdsx*^*FΔ85*^ (**i**), and normal male animals (**j**). The dotted line indicates the testis-like tissues

To get more insight into the gene expression profile in the gonads of *Masc-R*/+, *Bmdsx*^*MΔ7*/ *MΔ7*^ females, we performed qRT-PCR to quantify expression levels of *Bmovo-1* and *Bm-nosO*, both of which are important for oogenesis in the silkworm [24, 25]. While *Masc-R* expression in females suppressed the expression of these two genes (Fig. 1c and 3d), the expression levels of *Bmovo-1* and *Bm-nosO* were restored in *Masc-R* females homozygous for the *BmdsxM* mutation. These results further supported our previous findings that the egg production ability was restored in *Masc-R* females homozygous for the *BmdsxM* mutation (Fig. 2a and b).

Combined, these results demonstrated that *BmdsxM* depletion in *Masc-R* females restored the ability of egg production by inducing the expression of genes important for oogenesis and egg formation.

### *BmdsxF* depletion in *Masc*-*R* females promotes female to male sex reversal in internal and external genitalia

We performed similar analyses using females with forced *Masc-R* expression and lack of *BmdsxF* expression, which were generated by crossing the *Masc-R* strain with a *BmdsxF* deletion mutant strain that was established in this study (Additional files 6 and 10). This mutant strain had an 85-bp deletion in the female-specific *Bmdsx* exon (exon 3) and its adjacent intron sequence, resulting in no BmDSX protein production (Additional files 5 and 9). qRT-PCR analysis demonstrated that the homozygous mutation in *BmdsxF* abolished *BmdsxF* expression in the *Masc-R* females. Although *Masc-R* females expressed *BmdsxM* despite being female, *BmdsxM* expression levels were lower compared with *Masc-R* females heterozygous for the *BmdsxF* mutation (Fig. 3a and b). Importantly, *Masc-R* females homozygous for the *BmdsxF* mutation developed internal genitalia that consisted of male-specific accessory glands, seminal vesicles, vas deferens, and ejaculatory duct and whose shape resembled normal male genitalia (Fig. 3f and g). In addition, testes similar in morphology with testes from normal males were observed at the apical end of the vas deferens in *Masc-R*/+, *Bmdsx* ^*FΔ85*/ *FΔ85*^ females (Fig. 3i and j). However, unlike normal males, the apical end of the vas deferens in these females was divided into several tubes (Fig. 3g and Additional file 12).

In comparison, *Masc-R* females heterozygous for the *BmdsxF* mutation (genetically the same as the *Masc-R*/+ females) formed degenerated ovaries (Fig. 3e) that were consistent with those previously reported in *Masc-R* females [9]. Moreover, testis-like tissues were observed at the apical end of ovarioles (Fig. 3h). These results indicated that *BmdsxF* depletion in *Masc-R* females drives female to male sex reversal in the internal genitalia.

**Fig. 4:**
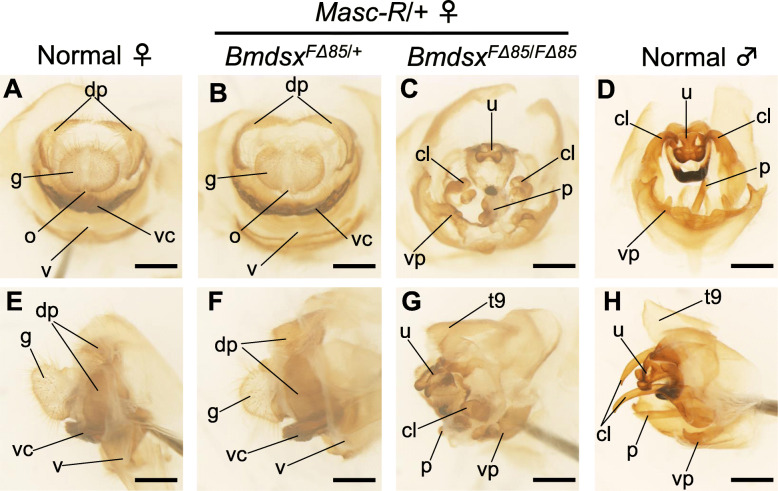
Morphological analysis of the external genitalia of *Masc-R* females homozygous for the *BmdsxF* mutation. The external genitalia were observed under a stereomicroscope. **a–d** Frontal and (**e–h**) lateral views of the external genitalia. **a, e** Normal females. **b, f** *Masc-R* females with heterozygous *Bmdsx*^*F∆85*^ mutation. **c, g** *Masc-R* females with homozygous *Bmdsx*^*F∆85*^ mutation. **d, h** Normal male. cl, clasper; dp, dorsal chitin plate; g, genital papilla; o, oviparous; p, penis; u, uncus; v, vagina; vc, ventral chitin plate; vp, ventral plate. Scale bars indicate 1 mm

**Fig. 5:**
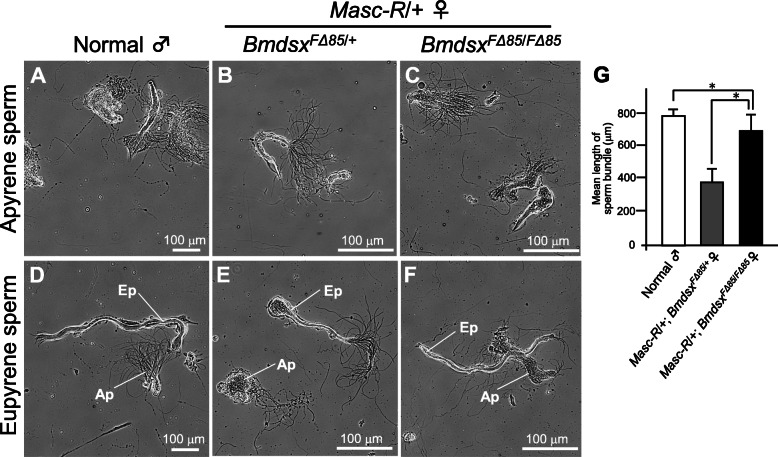
Morphological analysis of the sperm bundles of *Masc-R* females homozygous for *BmdsxF* mutation. The sperm bundles isolated from adult testes were observed under a phase-contrast microscope. **a** Apyrene sperm bundles from normal male animals. **b**, **c** The sperm bundles isolated from *Masc-R* females with *Bmdsx*^*FΔ85*^ heterozygous (**b**) or homozygous mutation (**c**) resembled apyrene sperm bundles. **d** Eupyrene sperm bundles from normal male animals. **e**, **f** The sperm bundles isolated from *Masc-R* females with *Bmdsx*^*FΔ85*^ heterozygous (**e**) or homozygous mutation (**f**) resembled eupyrene sperm bundles. Ap, apyrene sperm bundle; Ep, eupyrene sperm bundle. **g** The mean length of the sperm bundles. Error bars indicate standard deviation. * indicates a significant difference, as determined by Welch’s *t*-test

**Fig. 6:**
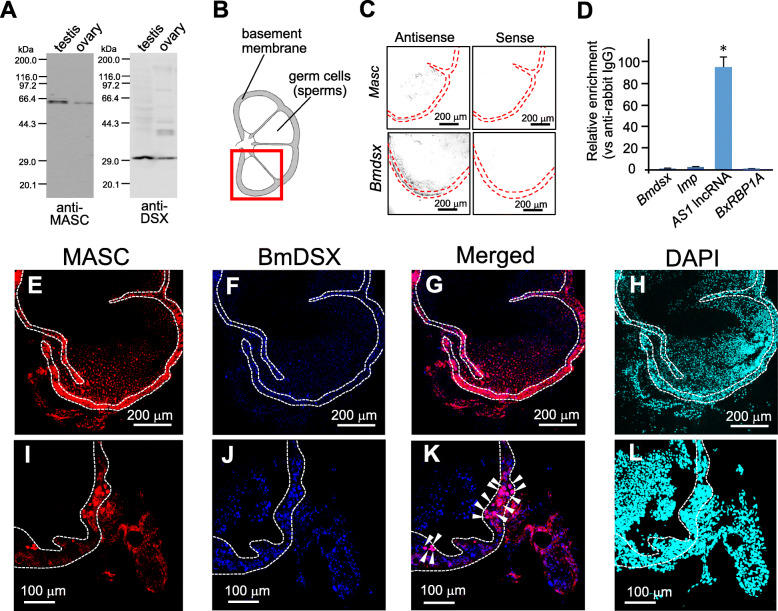
Development of an antibody targeting endogenous MASC protein and RIP-qPCR analysis. The specificity of the developed anti-MASC antibody was evaluated by western blotting and immunohistochemistry. **a** Western blottings using the anti-MASC antibody and an anti-DSX-DBD antibody used in the previous study [26] were performed with whole protein extracts from gonads of day-3 5th instar larvae. Sizes of the molecular markers are indicated on the left. Histone H3 protein levels were used as loading control (Additional file 9, right panel, lanes 1 and 2). **b** Cross-section illustration of the testis from a 5th instar larva. The testicular basement membrane is shown in gray. The tissue area shown in the immunostained image is indicated by a red box. **c** Frozen testis sections from day-3 5th instar larva were stained with a *Masc*-specific antisense riboprobe or with its sense strand probe (upper panel). The same ISH was performed using a *Bmdsx*-specific antisense riboprobeor its sense strand probe (lower panel). **d** RNA/MASC protein complexes were immunoprecipitated using the anti-MASC antibody, followed by qRT-PCR. The relative enrichment was defined as the level of qPCR product amplified from samples relative to that from samples precipitated using a negative control antibody (anti-rabbit IgG antibody). Values are presented as the mean ± SE from three independent qPCR assays. Results are representative of three independent experiments. * indicates a significant difference (< 0.05) compared with the negative control, as determined by Welch’s *t*-test. **e** Immunostaining using anti-MASC antibody was used to examine whether the antibody detected localization of the endogenous MASC protein in the testis. **f** The same section of the testis was subjected to immunostaining using an anti-DSX-DBD antibody. **g** Merged laser confocal microscopy image showing MASC (red) and BmDSX (blue) expression. **h** Nuclei were counterstained with DAPI. The dotted line indicates the testicular basement membrane. **i–l** High magnification images of testis sections immunostained with anti-MASC (**i**) or anti-DSX-DBD antibody (**j**). **k** Merged image showing MASC (red) and BmDSX (blue) expression. Arrowheads indicate MASC and BmDSX co-localization. (**l**) Nuclei were counterstained with DAPI

To extend our findings to other sexually dimorphic traits, we performed a morphological analysis of the external genitalia*.* Unlike similar previous studies of lepidopteran insects, we prepared cuticle specimens of the external genitalia, which enabled more accurate determination of the morphological changes in cuticle structures. The external genitalia of *Masc-R*/+ females heterozygous for the *BmdsxF* mutation had morphological characteristics similar to those of normal females (Fig. 4a–6d). In comparison, the external genitalia of *Masc-R* females homozygous for the *BmdsxF* mutation were malformed, with partial development of several male-specific genital organs, such as the uncus, clasper, penis, and 9th tergite, which is unique to males (Fig. 4e and f). The shape of the ventral plate was also similar to that of normal males. These results strongly support our previous findings that *BmdsxF* depletion in *Masc-R* females promotes maleness.

### *BmdsxF* knockout in *Masc*-*R* females enhances spermatogenesis

We next investigated whether the testis-like tissues and the testes found in *Masc-R* females heterozygous or homozygous for *BmdsxF* mutation have the ability to produce spermatozoa. In the silkworm, males produce two types of sperm bundles, one of which consists of eupyrene sperm and the other of which is composed of apyrene sperm [27, 28]. The testis-like tissues observed in *Masc-R* females heterozygous for the *BmdsxF* mutation contained sperm bundles that resembled apyrene sperm bundles (Fig. 5a and b). Similarly, the testes of *Masc-R* females homozygous for *BmdsxF* mutation contained apyrene sperm bundles (Fig. 5c). Although the testis-like tissues also contained sperm bundles that represented eupyrene sperm bundles, their size was smaller than that produced by normal male animals, and their shape was abnormal (Fig. 5d, e, and g). The testes of *Masc-R* females homozygous for *BmdsxF* mutation contained sperm bundles that resembled eupyrene sperm bundles, the size and shape of which were similar to those observed in male animals (Fig. 5d, f, and g). These results demonstrated that *BmdsxF* depletion, and thus the expression of *BmdsxM* alone, promoted spermatogenesis, although the genetic sex of germ cells were all ZW females.

### *BmdsxF* knockout in *Masc*-*R* females induces the expression of genes involved in spermatogenesis

To evaluate the spermatogenesis observed in *Masc-R*/+, *Bmdsx*^*F∆85/F∆85*^ females, we performed qRT-PCR to quantify the expression of *Bombyx* orthologs of the *Maelstrom* (*Mael*) and *always early* (*aly*) genes (designated *BmMael* and *BmAly*, respectively), which are important for spermatogenesis and meiotic progression and spermatid differentiation in the silkworm [29, 30]. Although the expression of these two genes in *Masc-R* females heterozygous for the *BmdsxF* mutation (genetically the same as *Masc-R*/+ females) was higher than that in normal females, the levels were still lower than those in normal males (Fig. 3c and d). The expression of *BmMaelstrom* and *BmAly* in *Masc-R* females homozygous for the *BmdsxF* mutation was significantly higher than in *Masc-R* females heterozygous for the *BmdsxF* mutation and were comparable to those in normal males (Fig. 3c and d). These results support the above findings that *BmdsxF* depletion in *Masc-R* females promotes spermatogenesis by increasing the expression of genes important for spermatogenesis (Fig. 4).

Combined, these results demonstrate that *BmdsxF* depletion in *Masc-R* females restored the ability to produce eggs by inducing the expression of genes important for spermatogenesis, meiotic progression, and spermatid differentiation.

### MASC protein interacts with the lncRNA from the *Bmdsx-AS1* gene

Our results revealed that *Masc-R* promotes the development of male characteristics in genitalia, including the gonads and the germ cells, by inducing the expression of *BmdsxM*. *Masc* is required for the male-specific splicing of *Bmdsx* transcripts, giving rise to *BmdsxM* [8, 10]. Therefore, we hypothesized that *Masc* might directly mediate male-specific *Bmdsx* splicing. To assess the potential interaction between *Masc* and *Bmdsx*, we performed RNA immunoprecipitation (RIP) in testis samples using a polyclonal antibody against MASC protein. Western blotting using whole protein extract from testes revealed that our anti-MASC antibody specifically recognized a protein with a molecular weight that was consistent with the putative molecular weight of the MASC protein (64.8 kDa, Fig. 6a, left panel). The same Western blotting with an anti-DSX-DBD antibody detected a single protein band with the putative molecular weight of the BmDSXM (30.0 kDa) and BmDSXF (29.5 kDa) proteins (Fig. 6a, right panel). Immunostaining using the anti-MASC antibody indicated that MASC protein was predominantly localized in cells at the testicular basement membrane (Fig. 6b, e and i). Similarly, immunostaining analysis using the anti-DSX-DBD antibody revealed that the BmDSX protein was also expressed in cells of the testicular basement membrane (Fig. 6f and j) and that it co-localized with MASC (Fig. 6g). These results were confirmed by in situ hybridization (ISH) using *Masc* or *Bmdsx*-specific riboprobes (Fig. 6c). Moreover, immunostaining demonstrated the co-localization of MASC and BmDSX in the cell nucleus (Fig. 6k, arrow heads).

The co-localization of MASC with BmDSX in the nucleus of cells found in the testicular basement membrane further supported the possibility that MASC may directly promote male-specific splicing in *Bmdsx*. To investigate whether MASC interacts with *Bmdsx* pre-mRNA, we performed RIP using the anti-MASC antibody, followed by qRT-PCR. Contrary to our expectation, we found no significant enrichment of MASC on *Bmdsx* pre-mRNA (Fig. 6d). Instead, specific binding of MASC on a long noncoding RNA transcribed from the *Bmdsx-AS1* gene (designated as *AS1* lincRNA), which is a testis-specific factor involved in the male-specific splicing of *Bmdsx*, was observed with the same analysis (Fig. 6d) [13]. Significant enrichment was not observed in any of the other RNAs examined, some of which were genes reportedly implicated in male-specific *Bmdsx* splicing. These results suggested the possibility that physical interaction between MASC and *AS1* lncRNA may be important for inducing the male-specific splicing of *Bmdsx* pre-mRNA*,* giving rise to *BmdsxM* in the testis.

## Discussion

In this study, we clarified the genetic relationship between *Masc* and *dsx* using a transgenic line and different mutant lines newly established by genome editing. To our knowledge, few studies have revealed the genetic interactions between multiple genes with such genetic analysis in insects other than *Drosophila*. We showed that *BmdsxM* depletion in *Masc-R* female silkworms fully restored ovary development (Fig. 1f and i) and female fertility (Fig. 2a and b), while *BmdsxF* knockout caused female to male sex reversal in the internal genitalia of *Masc-R* females (Fig. 3f and i). These results suggest that *Bmdsx* acts as a critical regulator of sexual differentiation in the internal and external genitalia of the silkworm, including the gonads and germ cells. Moreover, *Masc* acts as an intermediate regulator that shifts the sexual differentiation of the internal genitalia from female to male by promoting male-specific splicing in *Bmdsx* pre-mRNA. Although several studies have reported the functions of *Masc* and *dsx* in lepidopterans, they have only partly captured the effects of these two genes on sexually dimorphic traits. Our study is the first to elucidate comprehensively the role of *Masc* and *Bmdsx* in the sexual differentiation of a wide range of sexually dimorphic traits including germ cells, gonads, internal reproductive organs, and the external genitalia.

The expression of *BmdsxF* in *Masc-R* females homozygous for the *BmdsxM* mutation was significantly higher by seven-fold compared with *Masc-R* females heterozygous for the *BmdsxM* mutation. This is likely because the 7-bp deletion introduced in *Bmdsx* (*Bmdsx*^*MΔ7*^) had some effect on the sex-specific splicing of *Bmdsx* pre-mRNA. While *Bmdsx* undergoes female-specific splicing by default [31], the *Bmdsx*^*MΔ7*^ mutation may have impaired the efficiency of male-specific *Bmdsx* splicing, resulting in increased levels of *BmdsxF* mRNA production. This hypothesis is further supported by the finding that increased levels of *BmdsxF* expression were also observed in males homozygous for the *Bmdsx*^*MΔ7*^ mutation (Additional file 7).

In addition to *BmdsxF*, the expression of *Bmovo-1* and *Bm-nosO* was significantly increased in *Masc-R* females homozygous for the *Bmdsx*^*M∆7*^ mutation (Fig. 1c and d). These results suggest that *BmdsxF* positively regulates *Bmovo-1* and *Bm-nosO* expression, while *BmdsxM* suppresses their expression. Bmovo-1 protein is primarily found in the nuclei of ovarian cells and has been implicated in oogenesis. *Bmovo-1* knockdown resulted in degenerated ovaries and markedly fewer oocytes [24]. *Bm-nosO* is required for the arrangement of eggs in the ovariole and normal morphogenesis of eggs [25]. BmDSX proteins act as transcription factors regulating the expression of genes involved in the development of sexually dimorphic traits [15]. Consistent with previous findings, our immunostaining analysis using an anti-DSX antibody revealed the nuclear localization of BmDSX proteins (Fig. 6f and j). BmDSXF likely functions as a transcription factor to induce the expression of *Bmovo-1* and *Bm-nosO*, promoting egg development. There are no reports that *dsx* is involved in the regulation of *ovo* and *nanos* in other insect species, including *Drosophila*. Therefore, it is reasonable to consider that *Bmdsx* controls oogenesis through a pathway unique to the silkworm. Further studies should assess whether BmDSXF directly regulates the transcription of *Bmovo-1* and *Bm-nosO*.

The expression of *BmdsxM* in *Masc-R* females homozygous for the *BmdsxF* mutation was considerably lower compared with *Masc-R* females heterozygous for the same mutation (Fig. 3a). This reduction in *BmdsxM* expression in *Bmdsx*^*F∆85/∆85*^ females may have resulted from the 85-bp deletion (*Bmdsx*^*F∆85*^) in the female-specific alternative *Bmdsx* exon (exon 3) and its adjacent intronic sequence (Additional file 5). Intronic sequences flanking alternatively spliced exons function as intronic splicing enhancers or silencers (ISE or ISS), which enhance or repress the splice-site decision, respectively [32–34]. The 85-bp deletion may have affected the efficiency of male-specific splicing of *Bmdsx* pre-mRNA, leading to reduced levels of *BmdsxM* transcripts.

Despite the profoundly reduced *BmdsxM* expression levels, *Masc-R* females homozygous for the *Bmdsx*^*FΔ85*^ mutation developed internal genitalia whose morphology resembled normal male internal genitalia (Fig. 3f and g). The same homozygous mutation also caused a marked decrement in *Bmdsx* expression levels in males (Additional file 11); however, these animals did not show any abnormalities in genitalia and exhibited normal male fertility. It is reasonable to speculate that the expression levels of *BmdsxM* were sufficient to promote male development in the internal genitalia. Alternatively, loss of *BmdsxF* expression may have a higher effect on normal male development. As shown in Fig. 3b, the expression levels of *BmdsxF* in *Masc-R* females homozygous for the *Bmdsx*^*FΔ85*^ mutation were significantly lower compared with heterozygous females and similar to the expression levels observed in normal males. It has been reported that BmDSXF and BmDSXM compete with each other for target site binding when both are present [15]. In *D. melanogaster*, the final phenotype of male-specific morphological structures in males carrying the *hsp83-dsxF* transgene depended upon the ratio of DSXF and DSXM [35]. Thus, male development due to *BmdsxF* depletion would be more profound in *Masc-R* females homozygous for *Bmdsx*^*FΔ85*^ mutation compared with heterozygous females.

In addition to the masculinization observed in the internal genitalia, the sperm in *Masc-R* females homozygous for the *Bmdsx*^*FΔ85*^ mutation resembled normal male sperm morphologically to a greater extent than the sperm observed in females heterozygous for the same mutation (Fig. 5a–f). This was particularly remarkable for eupyrene sperm (Fig. 5f). These results are consistent with the finding that the expression of *BmMael* and *BmAly* in *Masc-R* females homozygous for *BmdsxF* mutation was significantly higher than in the *Masc-R* females heterozygous for the *BmdsxF* mutation (Fig. 3c and d). *BmMael*, which is a *Bombyx* ortholog of *Maelstrom*, regulates spermatogenesis in the silkworm [29]. *Maelstrom* is also essential for spermatogenesis in *Drosophila* and the mouse [36, 37]. The *D. melanogaster always early* gene (*DmAly*) is required for the onset of spermatid differentiation and maintenance of normal chromatin structure in primary spermatocytes [38, 39]. *BmAly* is a *Bombyx* ortholog of the *DmAly* gene and is important for meiotic progression and spermatid differentiation in the silkworm [30]. As is the case for the internal genitalia, the impact of *BmdsxF* depletion might be more important for male differentiation of germ cells than the presence of sufficient *BmdsxM* levels. It is possible that normal spermatogenesis is repressed in the presence of *BmdsxF* transcripts. There are no reports that loss of function of the female-specific isoform of *dsx* causes spermatogenesis in genetically female germ cells. This strongly suggests that *dsx* in the silkworm has a novel function different from that of *Drosophila dsx*.

However, the masculinization of several features in the genitalia and germ cells was still incomplete. Unlike wild-type males, the apical end of the vas deferens observed in the *Masc-R* females homozygous for *BmdsxF* mutation was divided into several tubes (Fig. 3i). Often the number of malformed tubes was four, which is consistent with the number of oviducts typically found in each ovary (Additional file 12). Thus, female to male sex reversal may have been incomplete in the apical end of the vas deferens, resulting in the formation of malformed tubes. Similar signs of incomplete male development were also observed in the external genitalia of *Masc-R* females homozygous for *BmdsxF* mutation, where partial formation of male genital organs, such as uncus and clasper, was observed, in addition to the presence of female genital organs (Fig. 4c and g). These intersexual phenotypes may have resulted from insufficient levels of *BmdsxM* expression. It is likely that male differentiation of the external genitalia is more dependent on *BmdsxM* than the internal genitalia.

Similar incomplete masculinization was also observed in sperm. The size of the eupyrene sperm bundles observed in *Masc-R* females homozygous for the *Bmdsx*^*F∆85*^ mutation was significantly smaller compared with wild-type male silkworms (Fig. 5f). The total number of sperm bundles appeared considerably lower (data not shown). These findings suggest that *Masc-R* females homozygous for the *Bmdsx*^*F∆85*^ mutation still lack certain factors vital for spermatogenesis. In *D. melanogaster*, in addition to sex determination signals from somatic cells, the sex chromosome constitution of the germ cells is important for germline sex determination. The presence of the Y chromosome is indispensable for spermatogenesis, as it contains certain genes that are essential for spermatogenesis. Similarly, the male chromosomal constitution (ZZ) may also be necessary for spermatogenesis in the silkworm. A significant number of testis-specific genes have already been mapped on the Z chromosome in the silkworm [40]. Such enrichment of testis-specifically expressed genes on the Z chromosome is closely related to the male-specific chromosomal constitution, where the male sex is determined by the two Z chromosomes [40]. Moreover, the expression of genes mapped on the Z chromosome in the testis was more than ten-fold higher on average than in the ovary [41]. Therefore, it is reasonable to speculate that the male chromosomal constitution (two Z chromosomes) in germ cells is essential for achieving complete spermatogenesis.

To our knowledge, this is the first study to demonstrate the interaction of MASC proteins with *AS1* lncRNA (Fig. 6d). Immunostaining analysis demonstrated that MASC was localized in the nucleus of cells found on the testicular basement membrane (Fig. 6e and i). This result is consistent with previous studies reporting that MASC contains a nuclear localization signal and exhibits nuclear localization in cultured cells expressing a *Masc* transgene [42]. *AS1* lncRNA expression was also observed in the nucleus of testicular cells [13]. These findings strongly suggest that MASC proteins bind to *AS1* lncRNA in the cell nucleus. The gene encoding *AS1* lncRNA is expressed in a testis-specific manner, and its knockdown in males shifts the splicing pattern of *Bmdsx* from male to female mode [13]. *AS1* lncRNA contains a nucleotide sequence complementary to *Bmdsx* pre-mRNA; hence, it is possible that *AS1* lncRNA interacts directly with *Bmdsx* pre-mRNA, inducing male-specific splicing. MASC proteins potentially interact with *AS1* lncRNA, augmenting its ability to induce male-specific *Bmdsx* splicing. Since no significant enrichment of MASC on *Bmdsx* pre-mRNA was observed (Fig. 6d), it is conceivable that MASC-*AS1* lincRNA interaction indirectly regulates the male-specific splicing of *Bmdsx* pre-mRNA. For example, MASC binding to *AS1* lncRNA could protect the lncRNA from degradation, resulting in enhanced *AS1* lncRNA-mediated male-specific *Bmdsx* splicing. How exactly the interaction between MASC and *AS1* lncRNA promotes male-specific splicing in *Bmdsx* pre-mRNA remains to be elucidated.

Our Western blotting analysis unexpectedly detected the weak but definite MASC expression in the ovary (Fig. 6a). In this regard, it has recently been reported that *Masc* has an important role in female differentiation of the external genitalia [43]. This suggests that *Masc* has some functions in female differentiation. Our study is the first example to suggest that *Masc* may have some functions for the female differentiation in the internal genitalia and or germ cells. This finding will be a great help to understand the novel features of *Masc*.

## Methods

### Silkworm maintenance, generation of *Bmdsx* knockout silkworms, and crossing

*Bombyx mori* strains were maintained under standard conditions [44]. The *Masc-R* strain, the generation of which was described in our previous study [9], was used in the present study. Knockout silkworms were generated using transcription activator-like effector nucleases (TALENs), as described previously [45, 46]. The target sites of TALENs within the female-specific exon (exon 3) and male-specific coding region in exon 5 are illustrated in Additional file 5. Generation 0 (G0) hatched larvae were reared to adults, and G0 adults were crossed. Mutant strains were established and maintained as described in our previous study [46]. Primer sequences and PCR conditions used for genotyping are shown in Additional file 1. Genomic PCR for genotyping was performed according to a previously described protocol [47]. The resulting mutant lines *Bmdsx*^*FΔ85*^ and *Bmdsx*^*MΔ7*^ harbored an 85-bp deletion and a 7-bp deletion within the target sites, respectively (Additional file 5). *Masc-R*/+ males were bred with females homozygous for the *Bmdsx*^*MΔ7*^ mutation, and *Masc-R*/+ females homozygous for the *Bmdsx*^*MΔ7*^ mutation were obtained according to the procedure illustrated in Additional file 6. *Masc-R*/+ females homozygous for the *Bmdsx*^*FΔ85*^ mutation were generated following the procedure described in Additional file 10. Wild-type females and males obtained from the same parent were served as normal female and male controls.

### RNA extraction and reverse transcription (RT)-PCR

Total RNA extraction from silkworm tissues using ISOGEN (Nippon Gene) and subsequent RT-PCR was performed as described previously [48]. The primer sequences and PCR conditions used for the RT-PCRs are shown in Additional file 2.

### Quantitative real-time RT-PCR (qRT-PCR)

qRT-PCR assays were performed according to a previously described protocol [45]. The primer sequences used for qRT-PCR are listed in Additional file 3. Amplification of elongation factor-2 (EF-2) as an internal control using the BmEF-2F1 and BmEF-2R1 primers was also performed for quantification [49].

### In situ hybridization (ISH)

Localization analysis of *Masc* and *Bmdsx* mRNAs using in situ hybridization (ISH) was performed as previously described [50, 51]. Digoxygenin-labeled sense and antisense riboprobes were synthesized using a DIG RNA labeling kit (Roche) and PCR-amplified cDNAs as a template, according to the manufacturer’s instructions. The PCR primer sequences used for ISH are listed in Additional file 4.

### Western blotting

Testes and ovaries from day-3 5th instar larvae were sonicated (two sets of 1-s pulse × 10 times) in 2× sample buffer (100 mM Tris-HCl [pH 6.8], 4% SDS, 12% β-mercaptoethanol), followed by incubation at 95 °C for 3 min. The resulting product was centrifuged (15,000 rpm, 25 °C, 5 min), and the supernatants were used for western blotting. Equal amounts (5 μg) of protein were separated by 12.5% SDS-PAGE and then transferred onto PVDF membranes. The membranes were immunoblotted with an anti-MASC polyclonal antibody (1:50 dilution) or an anti-DSX-DBD antibody (1:50 dilution) in blocking buffer (1× TBS-T containing 5% skimmed milk powder) overnight at 4 °C. The anti-MASC antibody was produced by Eurofins Genomics. The C-terminal fragment of MASC (CASKERKPEARNTEI), which was predicted to be the most appropriate epitope, was synthesized and used to immunize a rabbit. The anti-DSX-DBD monoclonal antibody was purchased from Developmental Studies Hybridoma Bank. HRP-conjugated mouse anti-rabbit IgG (1:3000; sc-2357, Santa Cruz Biotechnology) and an HRP-conjugated goat anti-mouse IgG-HRP (1:3000; sc-2005, Santa Cruz Biotechnology) were used as secondary antibodies. Using a detection reagent (Immunostar LD, Wako), the chemiluminescence signal on the membranes was detected on an ImageQuant LAS4000 system (GE Healthcare).

### Immunohistochemistry

Frozen sections were prepared using a cryostat and then fixed with acetone at − 20 °C. The sections were washed three times for 5 min with 1× TBS (pH 7.5), and then incubated in blocking buffer (1× TBS-T containing 5% normal goat serum) for 1 h at room temperature. After blocking, the sections were incubated either with anti-MASC antibody (1:10 dilution) or anti-DSX-DBD antibody (1:10 dilution) overnight at 4 °C. After three washes with 1× TBS (pH 7.5) for 5 min, the sections were incubated with a secondary antibody according to the protocol described previously [52]. Fluorescence images were acquired using the confocal laser scanning microscope FV3000 (Olympus).

### RNA Immunoprecipitation qPCR (RIP-qPCR)

The RIP-qPCR analysis was performed according to a previously described protocol with minor modifications [53]. Briefly, RIP samples were prepared from ten pooled testes of day-3 5th instar larvae using the anti-MASC antibody (12 μg per reaction). RNAs were extracted from the RIP samples using ISOGEN followed by treatment with TURBO DNase (Thermo Fisher Scientific), according to the manufacturer’s instructions. To calculate the amount of target sequence in the precipitated RNA/protein complexes, we performed qRT-PCR, as previously described [53]. The primer sequences used for RIP-qPCR are shown in Additional file 3.

## Supplementary information

**Additional file 1: Table S1.** Primer sequences and PCR conditions used for genotyping.

**Additional file 2: Table S2.** Primer sequences and PCR conditions used for RT-PCR.

**Additional file 3: Table S3.** Sequences of primers used for qRT-PCR.

**Additional file 4: Table S4.** Sequences of primers used to prepare riboprobes for ISH.

**Additional file 5: Fig. S1.** Generation of *BmdsxM* and *BmdsxF* knockout silkworm lines using TALENs. (A) The target sites of TALENs within the female-specific exon (exon 3) and the male-specific coding region in exon 5 are shown. The rectangles indicate exons. Exons 3 and 4 are skipped when *Bmdsx* pre-mRNA is spliced in males. The gray region encodes the female-specific open reading frame (ORF). The black region encodes the male-specific ORF. TAL effector-binding sequences are shown in blue, while spacer sequences are indicated in red. (B) The deletion mutations introduced in the *Bmdsx*^*FΔ85*^ and *Bmdsx*^*MΔ7*^ lines are shown in (B) and (C), respectively. The uppercase characters in (B) represent the nucleotide sequence of *Bmdsx* exon 3. The uppercase characters in (C) indicate the nucleotide sequence of *Bmdsx* exon 5. Colons indicate identical nucleotide sequences between wild-type and mutant animals. Spacer sequences are indicated in red. 5′ splice donor and 3′ splice acceptor sites are shown in bold characters.

**Additional file 6: Fig. S2.** Procedure followed to generate *Masc-R*/+ females homozygous for the *Bmdsx*^*MΔ7*^ mutation. The *Masc-R*/+ females homozygous for *Bmdsx*^*MΔ7*^ were generated by crossing *Masc-R* and *Bmdsx*^*MΔ7*^ animals. (A) In generation 0 (G0), *Masc-R*/+ males were crossed with females homozygous for the *Bmdsx*^*MΔ7*^ mutation. In the next generation (G1), animals heterozygous for the *Bmdsx*^*MΔ7*^ mutation were selected after PCR-based genotyping, and females without the *Masc-R* transgene were crossed with *Masc-R*/+ males. Individuals carrying the *Masc-R* transgene were selected based on the expression of the *egfp* marker gene, as described previously [9]. In the resulting offspring (G2), *Masc-R*/+ females homozygous for the *Bmdsx*^*MΔ7*^ mutation were subjected to further analyses. Individuals heterozygous for the *Bmdsx*^*MΔ7*^ mutation or individuals with wild-type *BmdsxM* were used as controls. (B) PCR-based genotyping for the identification of individuals homozygous or heterozygous for the *Bmdsx*^*MΔ7*^ mutation. Genomic PCR was performed as described in Materials and Methods, and the amplified product was separated by 10% polyacrylamide gel electrophoresis. The gels were stained with 1% ethidium bromide in 1× TAE buffer to visualize the DNA. The upper bands represent amplicons from wild-type *Bmdsx* animals, while the lower bands represent amplicons from *Bmdsx*^*MΔ7*^ mutants.

**Additional file 7: Fig. S3.** *Bmdsx* mRNA levels in *Bmdsx*^*FΔ85*^ and *Bmdsx*^*MΔ7*^ silkworms. Expression levels of *Bmdsx* at the mRNA level in the *Bmdsx* mutant lines used in this study were analyzed by qRT-PCR. *BmdsxM* mRNA levels in the internal genitalia of *Bmdsx*^*MΔ7*^ (A) and *Bmdsx*^*FΔ85*^ mutant animals (C), as determined by qRT-PCR. Similarly, the mRNA level of *BmdsxF* was quantified by qRT-PCR in *Bmdsx*^*MΔ7*^ (B) and *Bmdsx*^*FΔ85*^ mutants (D). Error bars indicate standard deviation. * indicates a significant difference, as determined by Welch’s *t*-test.

**Additional file 8: Fig. S4.** Expression pattern of *Bmdsx* was analyzed by RT-PCR using primers that can amplify both *BmdsxF* and *BmdsxM* transcripts at the same time. Template cDNAs were prepared from the internal genitalia of adults with indicated genotypes. The amplified product was separated by 10% polyacrylamide gel electrophoresis. The gels were stained with 1% ethidium bromide in 1× TAE buffer to visualize the DNA. The arrows indicate the DNA bands corresponding to the size of *BmdsxF*, *BmdsxM*, and *Bmdsx* ^*FΔ85*^ transcripts.

**Additional file 9: Fig. S5.** BmDSX protein levels in *Bmdsx*^*FΔ85*^ and *Bmdsx*^*MΔ7*^ lines. BmDSX protein levels were determined by western blotting using an anti-DSX-DBD antibody (left panel). Whole protein extracts from testes or ovaries of day-3 5th instar larvae with the indicated genotype were separated by 12.5% SDS-PAGE. The sizes of the molecular markers are indicated on the left. The arrow indicates the protein band corresponding to the molecular weight of each BmDSX protein. The expected molecular weights were as follows: BmDSXM, 32 kDa; BmDSXF, 29.5 kDa; BmDSX^MΔ7^, 26.6 kDa; BmDSX^FΔ85^, 24.8 kDa. Histone H3 protein levels were used as loading control (right panel).

**Additional file 10: Fig. S6.** Procedure followed to generate *Masc-R*/+ females homozygous for the *Bmdsx*^*FΔ85*^ mutation. *Masc-R*/+ females homozygous for *Bmdsx*^*FΔ85*^ were generated by crossing *Masc-R* and *Bmdsx*^*FΔ85*^ animals. (A) In generation 0 (G0), *Masc-R*/+ females were crossed with males homozygous for the *Bmdsx*^*FΔ85*^ mutation. In the next generation (G1), animals heterozygous for the *Bmdsx*^*FΔ85*^ mutation were selected after PCR-based genotyping, and females without the *Masc-R* transgene were crossed with *Masc-R*/+ males. Individuals carrying the *Masc-R* transgene were selected based on the expression of the *egfp* marker gene, as described previously [9]. In the resulting offspring (G2), *Masc-R*/+ females homozygous for the *Bmdsx*^*FΔ85*^ mutation were subjected to further analyses. Individuals heterozygous for the *Bmdsx*^*FΔ85*^ mutation or individuals with wild-type *BmdsxF* were used as controls. (B) PCR-based genotyping for the identification of individuals homozygous or heterozygous for the *Bmdsx*^*FΔ85*^ mutation. Genomic PCR was performed as described in Materials and Methods, and the amplified product was separated by 2% agarose gel electrophoresis. The gels were stained with 1% ethidium bromide in 1× TAE buffer to visualize the DNA. The upper bands represent amplicons from wild-type *Bmdsx*, while the lower bands represent amplicons derived from *Bmdsx*^*FΔ85*^ animals.

**Additional file 11: Fig. S7.** Expression levels of *BmdsxM* in males homozygous for the *Bmdsx*^*FΔ85*^ mutation. Expression levels of *BmdsxM* mRNA in males homozygous for the *Bmdsx*^*FΔ85*^ mutation were determined by qRT-PCR. Error bars represent standard deviation. * indicates a significant difference, as determined by Welch’s *t*-test.

**Additional file 12: Fig. S8.** Malformed tubes observed at the apical end of the vas deferens in *Masc-R* females homozygous for the *BmdsxF* mutation. Images around the apical end of the vas deferens were acquired by a digital camera attached to a stereomicroscope. (A) Normal male. (B–E) *Masc-R* females homozygous for the *Bmdsx* ^*FΔ85*^ mutation. The dotted lines indicate malformed tubes. T: testis, VD: vas deferens.

## Data Availability

All of the mutant lines established in this study are continuously reared and passaged at the Laboratory of Bio-resource Regulation, Department of Integrated Biosciences, Graduate School of Frontier Sciences, The University of Tokyo and Genetically Modified Organism Research Center, National Institute of Agrobiological Sciences. All data obtained or analysed during the present study are available from the corresponding author on reasonable request.

## Abbreviations

*Masc*: *Masculinizer*
*dsx*: *Doublesex*
*Bmdsx*: *Bombyx mori doublesex*
*BmdsxM*: Male isoform of *Bmdsx*
*BmdsxF*: Female isoform of *Bmdsx*
RIP: RNA immunoprecipitation
lncRNA: Long non-coding RNA
ORF: Open reading frame
RT-PCR: RNA extraction and reverse transcription-PCR
qRT-PCR: Quantitative real-time RT-PCR
*Bmovo-1*: *Bombyx mori ovo-1*
*Bm-nosO*: *Bombyx mori nanos* ortholog
*BmMaelstrom*: *Bombyx mori Maelstrom*
*BmAly*: *Bombyx mori always early* (*Aly*)
*Bmdsx*-*AS1*: *Bmdsx-anti-sense RNA 1*
mRNA: Messenger RNA
SDS: Sodium dodecyl sulfate
PAGE: Polyacrylamide gel electrophoresis
PBS: Phosphate-buffered saline
TAE: Tris-acetate-EDTA buffer
