# Supplementary material for: Transgenic and knockout analyses of Masculinizer and doublesex illuminated the unique functions of doublesex in germ cell sexual development of the silkworm, Bombyx mori
Source: BMC Dev Biol. 2020 Sep 21;20:19. doi: 10.1186/s12861-020-00224-2 (PMC7504827; doi:10.1186/s12861-020-00224-2)
Supplement: Supplementary file 1 — Additional file 1: Table S1. Primer sequences and PCR conditions used for genotyping. [file 12861_2020_224_MOESM1_ESM.pdf]

Supplementary Table1. Primer sequences and PCR conditions used for genotyping

Gene	Primers	Sequence	Denaturation	Annealing	Elongation	N°cycles
BmdsxMΔ7	dsxExon5s-1F	GACTGAAACGTCCGGACCG	98℃	57℃	72℃	30
	dsxExon5s-1R	GACAACTCCAGCGCTCCG	10 s	30 s	60 s	
BmdsxMΔ85	BmDSX-5F	AATACGTAACAGTGTTGCCAGTTG	98℃	57℃	72℃	30
	BmDSX-6R	GCCTTGAATGTACGTACGACGTGTC	10 s	30 s	60 s	
