# Supplementary material for: Transgenic and knockout analyses of Masculinizer and doublesex illuminated the unique functions of doublesex in germ cell sexual development of the silkworm, Bombyx mori
Source: BMC Dev Biol. 2020 Sep 21;20:19. doi: 10.1186/s12861-020-00224-2 (PMC7504827; doi:10.1186/s12861-020-00224-2)
Supplement: Supplementary file 2 — Additional file 2: Table S2. Primer sequences and PCR conditions used for RT-PCR. [file 12861_2020_224_MOESM2_ESM.docx]

**Supplementary Table 2.** Primer sequences and PCR conditions used for RT-PCR

| Gene | Primers | Sequence | Denaturation | Annealing | Elongation | N°**c**ycles |
| --- | --- | --- | --- | --- | --- | --- |
| *Bmdsx* | FF2 | CGCCTTACCGCAGACAGGCAG | 98℃ | 57℃ | 72℃ | 35 |
|  | FR4 | GCGCAGTGTCGTCGCTACAAGG | 10 s | 30 s | 60 s |  |
| *BmdsxMΔ7* | dsxExon5s-1F | GACTGAAACGTCCGGACCG | 98℃ | 57℃ | 72℃ | 35 |
|  | dsxExon5s-1R | GACAACTCCAGCGCTCCG | 10 s | 30 s | 60 s |  |
| *BmdsxMΔ85* | BmDSX-5F | AATACGTAACAGTGTTGCCAGTTG | 98℃ | 57℃ | 72℃ | 35 |
|  | BmDSX-6R | GCCTTGAATGTACGTACGACGTGTC | 10 s | 30 s | 60 s |  |
| *BmActin3* | BmA3-F | AGATGACCCAGATCATGTTCG | 98℃ | 55℃ | 72℃ | 35 |
|  | BmA3-R | GAGATCCACATCTGTTGGAAG | 10 s | 30 s | 60 s |  |
