# Supplementary material for: Transgenic and knockout analyses of Masculinizer and doublesex illuminated the unique functions of doublesex in germ cell sexual development of the silkworm, Bombyx mori
Source: BMC Dev Biol. 2020 Sep 21;20:19. doi: 10.1186/s12861-020-00224-2 (PMC7504827; doi:10.1186/s12861-020-00224-2)
Supplement: Supplementary file 3 — Additional file 3: Table S3. Sequences of primers used for qRT-PCR. [file 12861_2020_224_MOESM3_ESM.pdf]

Supplementary Table 3. Sequences of primers used for qRT-PCR

Gene	Primers	Sequence
BmdsxM	BmdsxM-F	CCGCCAACCATGCCACCACTG
	E2E5-R	TGCATCATCCAATAACCTTCG
BmdsxF	BmdsxF-F	AACTCGACACGCCAGAAAATG
	E4E5-R	GCATCATCCAATAACCCATAG
Masc	Masc RT-1F	GCTAAAATTGCTGGGATTGCTA
	Masc RT-1R	CAGCAATCGGAATTTTCTTCTG
Bmnanos	Bmnanos-F	GGATCCCGCTCCAAATAGCA
	Bmnanos-R	ATCGAATAGCGGGTCAGTGC
Bmovo1	Bmovo1-F	GCCCCTTACCGCTCCTTTCG
	Bmovo1-R	ATCGCCTCCAAGAATCGATG
α-tubulin	Tub-alpha-F	CCCGCGAAAAGGACAATACA
	Tub-alpha-R	ACGGTTGTAGGTGGCTGATA
Imp	IMPE7-F1	ATGCGGGAAGAAGGTTTTATG
	IMPE7-R1	TAATGTGAACGGTGGTCACGTG
BxRBP3A	BxRBP3A-F	GATGGAGATACAAGATTACTCC
	BxRBP3A and B-R	ACATCTGCCTGAGGTCATTCTC
AS1	AS1 lncRNA-F	TTCGTACTGGCTCTCTTCTCGT
	AS1 lncRNA-R	CAAAGTTGATAGCAATTCCCT
