# Supplementary material for: Transgenic and knockout analyses of Masculinizer and doublesex illuminated the unique functions of doublesex in germ cell sexual development of the silkworm, Bombyx mori
Source: BMC Dev Biol. 2020 Sep 21;20:19. doi: 10.1186/s12861-020-00224-2 (PMC7504827; doi:10.1186/s12861-020-00224-2)
Supplement: Supplementary file 4 — Additional file 4: Table S4. Sequences of primers used to prepare riboprobes for ISH. [file 12861_2020_224_MOESM4_ESM.pdf]

Supplementary Table 4. Sequences of primers for preparing riboprobes utilized in ISH

Gene	Primers	Sequence
Masc	Masc ISH probe-F	CACCACCCGTAGCATCAGTG
	Masc ISH probe-R	TTCGCGTCATCCAGTTTTGC
	Masc ISH-T7F	CCGGATCCTAATACGACTCACTATAGGGCGCACCACCCGTAGCATCAGTG
	Masc ISH- T7R	CCGGATCCTAATACGACTCACTATAGGGCGTTCGCGTCATCCAGTTTTGC
BmdsxM	BmdsxM ISH probe-F	AACTCGACACGCCAGAAAATG
	BmdsxM ISH probe-R	GCATCATCCAATAACCCATAG
	BmdsxM ISH-T7F1	CCGGATCCTAATACGACTCACTATAGGGCGCACCTGGGGTGTCACCATAC
	BmdsxM ISH-T7R1	CCGGATCCTAATACGACTCACTATAGGGCGATCTCGGAGGCAACATAGCG
