# Supplementary material for: Transgenic and knockout analyses of Masculinizer and doublesex illuminated the unique functions of doublesex in germ cell sexual development of the silkworm, Bombyx mori
Source: BMC Dev Biol. 2020 Sep 21;20:19. doi: 10.1186/s12861-020-00224-2 (PMC7504827; doi:10.1186/s12861-020-00224-2)
Supplement: Supplementary file 5 — Additional file 5: Fig. S1. Generation of BmdsxM and BmdsxF knockout silkworm lines using TALENs. (A) The target sites of TALENs within the female-specific exon (exon 3) and the male-specific coding region in exon 5 are shown. The rectangles indicate exons. Exons 3 and 4 are skipped when Bmdsx pre-mRNA is spliced in males. The gray region encodes the female-specific open reading frame (ORF). The black region encodes the male-specific ORF. TAL effector-binding sequences are shown in blue, while spacer sequences are indicated in red. (B) The deletion mutations introduced in the BmdsxFΔ85 and BmdsxMΔ7 lines are shown in (B) and (C), respectively. The uppercase characters in (B) represent the nucleotide sequence of Bmdsx exon 3. The uppercase characters in (C) indicate the nucleotide sequence of Bmdsx exon 5. Colons indicate identical nucleotide sequences between wild-type and mutant animals. Spacer sequences are indicated in red. 5′ splice donor and 3′ splice acceptor sites are shown in bold characters. [file 12861_2020_224_MOESM5_ESM.pptx]

## Slide 1
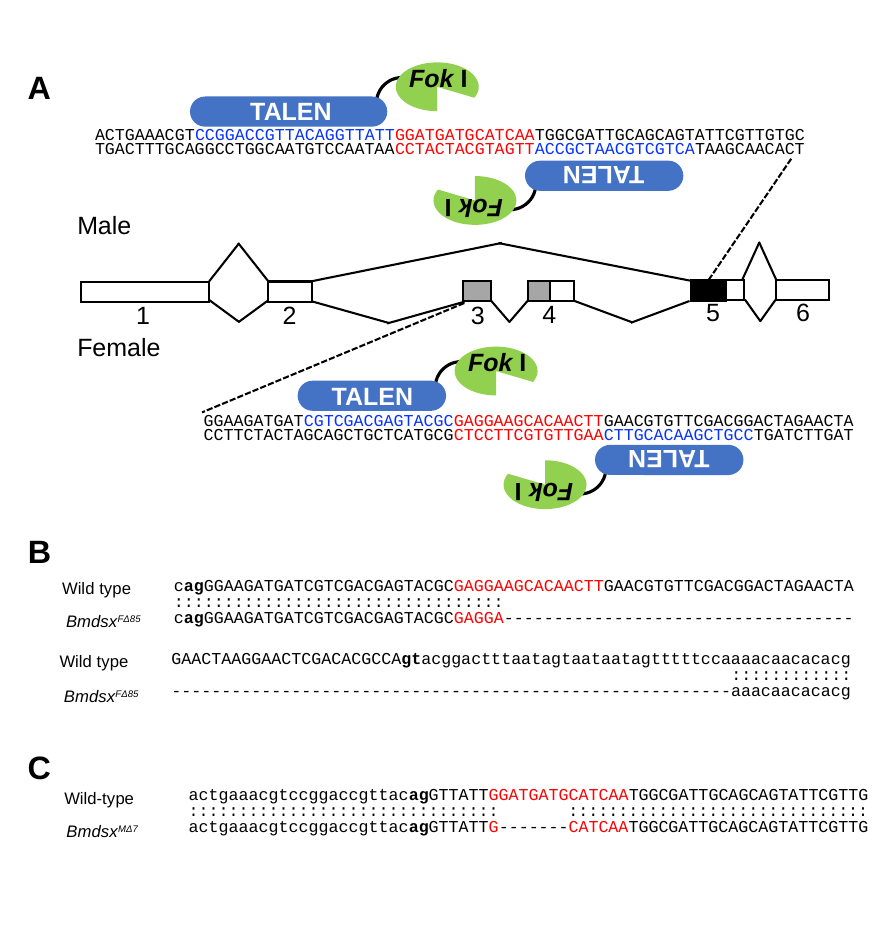

Fok I
TALEN
A
ActgaaacgtccggaccgttacagGTTATTGGATGATGCATCAATGGCGATTGCAGCAGTATTCGTTGTGC
TGACTTTGCAGGCCTGGCAATGTCCAATAACCTACTACGTAGTTACCGCTAACGTCGTCATAAGCAACACT
Fok I
TALEN
Male
5
6
4
3
1
2
Female
Fok I
TALEN
GGAAGATGATCGTCGACGAGTACGCGAGGAAGCACAACTTGAACGTGTTCGACGGACTAGAACTA
CCTTCTACTAGCAGCTGCTCATGCGCTCCTTCGTGTTGAACTTGCACAAGCTGCCTGATCTTGAT
Fok I
TALEN
B
Wild type
cagGGAAGATGATCGTCGACGAGTACGCGAGGAAGCACAACTTGAACGTGTTCGACGGACTAGAACTA
:::::::::::::::::::::::::::::::::
cagGGAAGATGATCGTCGACGAGTACGCGAGGA-----------------------------------
BmdsxFΔ85
Wild type
GAACTAAGGAACTCGACACGCCAgtacggactttaatagtaataatagtttttccaaaacaacacacg
 ::::::::::::
--------------------------------------------------------aaacaacacacg
BmdsxFΔ85
C
Wild-type
actgaaacgtccggaccgttacaggttattggatgatgcatcaatggcgattgcagcagtattcgttg
::::::::::::::::::::::::::::::: ::::::::::::::::::::::::::::::
actgaaacgtccggaccgttacaggttattg-------catcaatggcgattgcagcagtattcgttg
BmdsxMΔ7
