# Supplementary material for: Transgenic and knockout analyses of Masculinizer and doublesex illuminated the unique functions of doublesex in germ cell sexual development of the silkworm, Bombyx mori
Source: BMC Dev Biol. 2020 Sep 21;20:19. doi: 10.1186/s12861-020-00224-2 (PMC7504827; doi:10.1186/s12861-020-00224-2)
Supplement: Supplementary file 6 — Additional file 6: Fig. S2. Procedure followed to generate Masc-R/+ females homozygous for the BmdsxMΔ7 mutation. The Masc-R/+ females homozygous for BmdsxMΔ7 were generated by crossing Masc-R and BmdsxMΔ7 animals. (A) In generation 0 (G0), Masc-R/+ males were crossed with females homozygous for the BmdsxMΔ7 mutation. In the next generation (G1), animals heterozygous for the BmdsxMΔ7 mutation were selected after PCR-based genotyping, and females without the Masc-R transgene were crossed with Masc-R/+ males. Individuals carrying the Masc-R transgene were selected based on the expression of the egfp marker gene, as described previously [9]. In the resulting offspring (G2), Masc-R/+ females homozygous for the BmdsxMΔ7 mutation were subjected to further analyses. Individuals heterozygous for the BmdsxMΔ7 mutation or individuals with wild-type BmdsxM were used as controls. (B) PCR-based genotyping for the identification of individuals homozygous or heterozygous for the BmdsxMΔ7 mutation. Genomic PCR was performed as described in Materials and Methods, and the amplified product was separated by 10% polyacrylamide gel electrophoresis. The gels were stained with 1% ethidium bromide in 1× TAE buffer to visualize the DNA. The upper bands represent amplicons from wild-type Bmdsx animals, while the lower bands represent amplicons from BmdsxMΔ7 mutants. [file 12861_2020_224_MOESM6_ESM.pptx]

## Slide 1
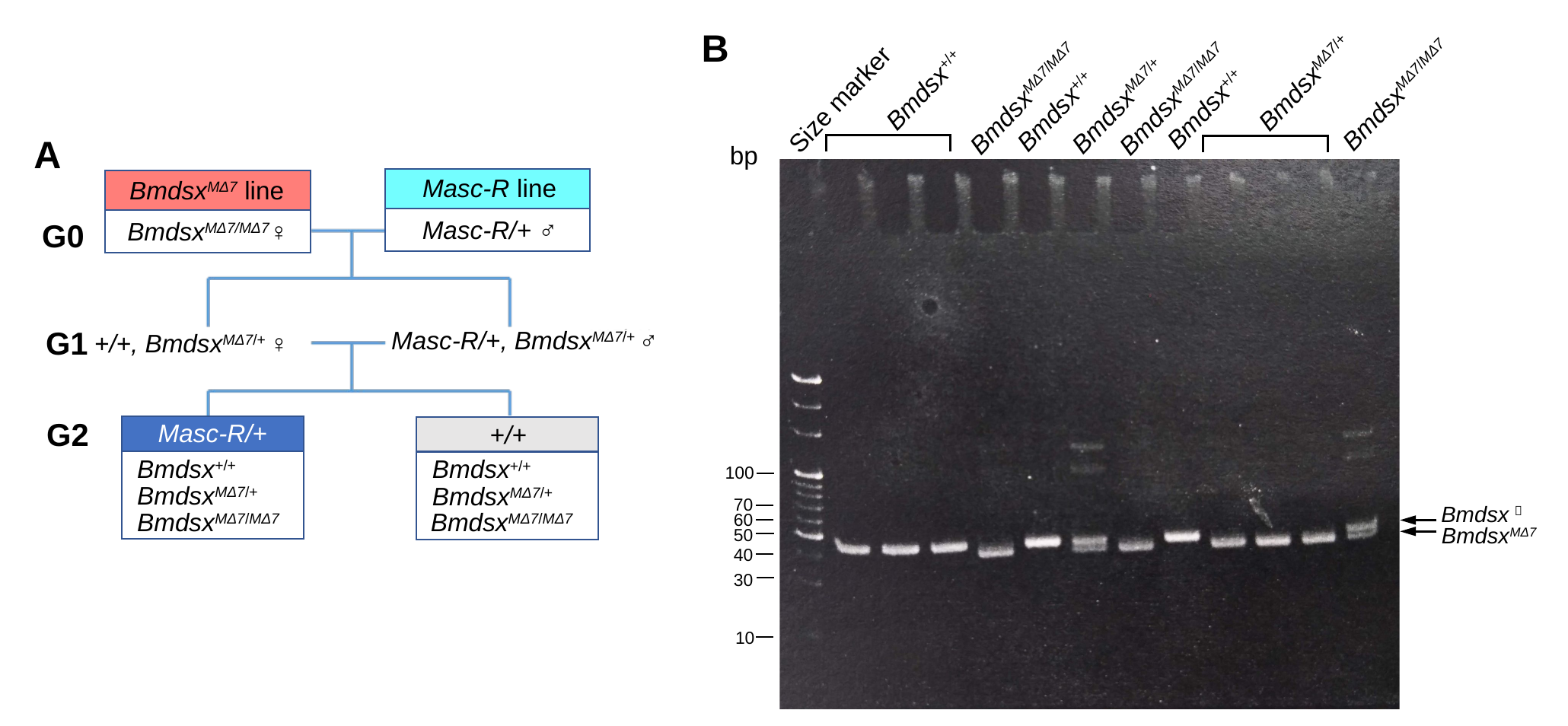

B
BmdsxMΔ7/MΔ7
Bmdsx+/+
BmdsxMΔ7/+
BmdsxMΔ7/MΔ7
BmdsxMΔ7/MΔ7
Bmdsx+/+
Bmdsx+/+
Size marker
BmdsxMΔ7/+
A
bp
Masc-R line
BmdsxMΔ7 line
Masc-R/+ ♂
G0
BmdsxMΔ7/MΔ7♀
G1
Masc-R/+, BmdsxMΔ7/+ ♂
+/+, BmdsxMΔ7/+ ♀
G2
Masc-R/+
+/+
Bmdsx+/+
BmdsxMΔ7/+
BmdsxMΔ7/MΔ7
Bmdsx+/+
100
BmdsxMΔ7/+
70
Bmdsx＋
BmdsxMΔ7/MΔ7
60
BmdsxMΔ7
50
40
30
10
