# Supplementary material for: Transgenic and knockout analyses of Masculinizer and doublesex illuminated the unique functions of doublesex in germ cell sexual development of the silkworm, Bombyx mori
Source: BMC Dev Biol. 2020 Sep 21;20:19. doi: 10.1186/s12861-020-00224-2 (PMC7504827; doi:10.1186/s12861-020-00224-2)
Supplement: Supplementary file 7 — Additional file 7: Fig. S3. Bmdsx mRNA levels in BmdsxFΔ85 and BmdsxMΔ7 silkworms. Expression levels of Bmdsx at the mRNA level in the Bmdsx mutant lines used in this study were analyzed by qRT-PCR. BmdsxM mRNA levels in the internal genitalia of BmdsxMΔ7 (A) and BmdsxFΔ85 mutant animals (C), as determined by qRT-PCR. Similarly, the mRNA level of BmdsxF was quantified by qRT-PCR in BmdsxMΔ7 (B) and BmdsxFΔ85 mutants (D). Error bars indicate standard deviation. * indicates a significant difference, as determined by Welch’s t-test. [file 12861_2020_224_MOESM7_ESM.pptx]

## Slide 1
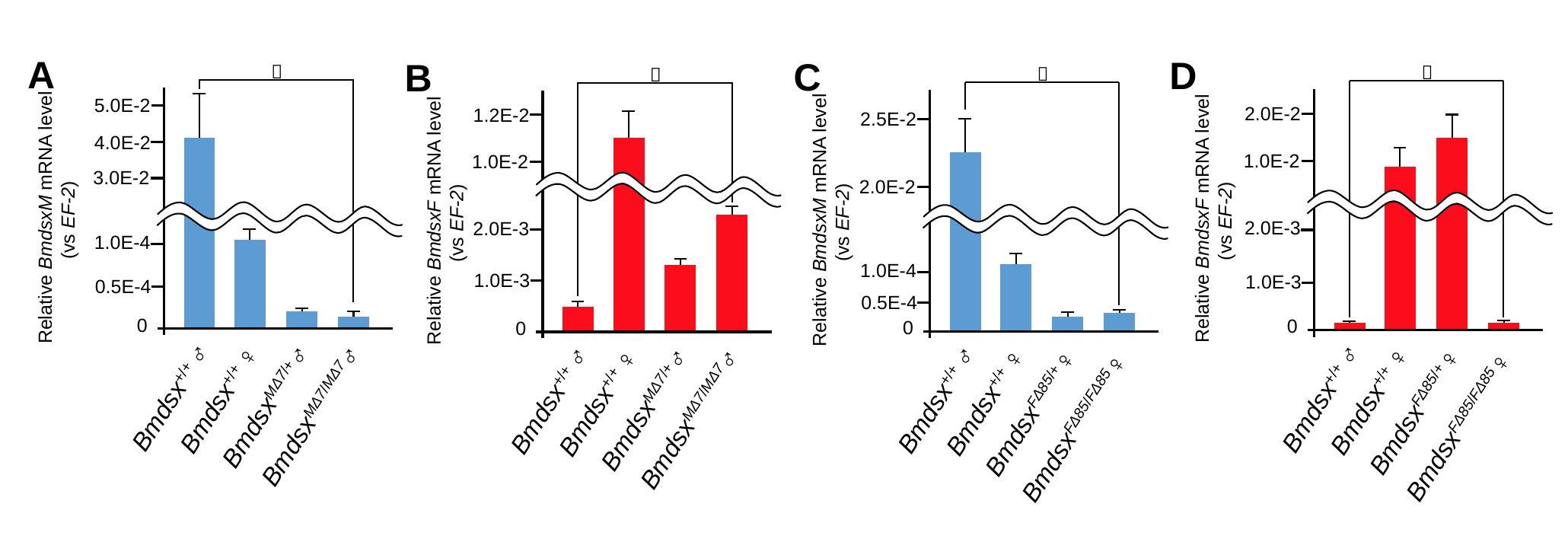

A
＊
5.0E-2
4.0E-2
3.0E-2
 Relative BmdsxM mRNA level
(vs EF-2)
1.0E-4
0.5E-4
0
BmdsxMΔ7/+♂
Bmdsx+/+ ♂
Bmdsx+/+ ♀
BmdsxMΔ7/MΔ7♂
D
C
＊
2.5E-2
2.0E-2
 Relative BmdsxM mRNA level
(vs EF-2)
1.0E-4
0.5E-4
0
Bmdsx+/+ ♂
Bmdsx+/+ ♀
BmdsxFΔ85/+♀
BmdsxFΔ85/FΔ85♀
B
＊
1.2E-2
1.0E-2
 Relative BmdsxF mRNA level
(vs EF-2)
2.0E-3
1.0E-3
0
BmdsxMΔ7/+♂
Bmdsx+/+ ♂
Bmdsx+/+ ♀
BmdsxMΔ7/MΔ7♂
＊
2.0E-2
1.0E-2
 Relative BmdsxF mRNA level
(vs EF-2)
2.0E-3
1.0E-3
0
Bmdsx+/+ ♂
Bmdsx+/+ ♀
BmdsxFΔ85/+♀
BmdsxFΔ85/FΔ85♀
