# Supplementary material for: Transgenic and knockout analyses of Masculinizer and doublesex illuminated the unique functions of doublesex in germ cell sexual development of the silkworm, Bombyx mori
Source: BMC Dev Biol. 2020 Sep 21;20:19. doi: 10.1186/s12861-020-00224-2 (PMC7504827; doi:10.1186/s12861-020-00224-2)
Supplement: Supplementary file 8 — Additional file 8: Fig. S4. Expression pattern of Bmdsx was analyzed by RT-PCR using primers that can amplify both BmdsxF and BmdsxM transcripts at the same time. Template cDNAs were prepared from the internal genitalia of adults with indicated genotypes. The amplified product was separated by 10% polyacrylamide gel electrophoresis. The gels were stained with 1% ethidium bromide in 1× TAE buffer to visualize the DNA. The arrows indicate the DNA bands corresponding to the size of BmdsxF, BmdsxM, and Bmdsx FΔ85 transcripts. [file 12861_2020_224_MOESM8_ESM.pptx]

## Slide 1
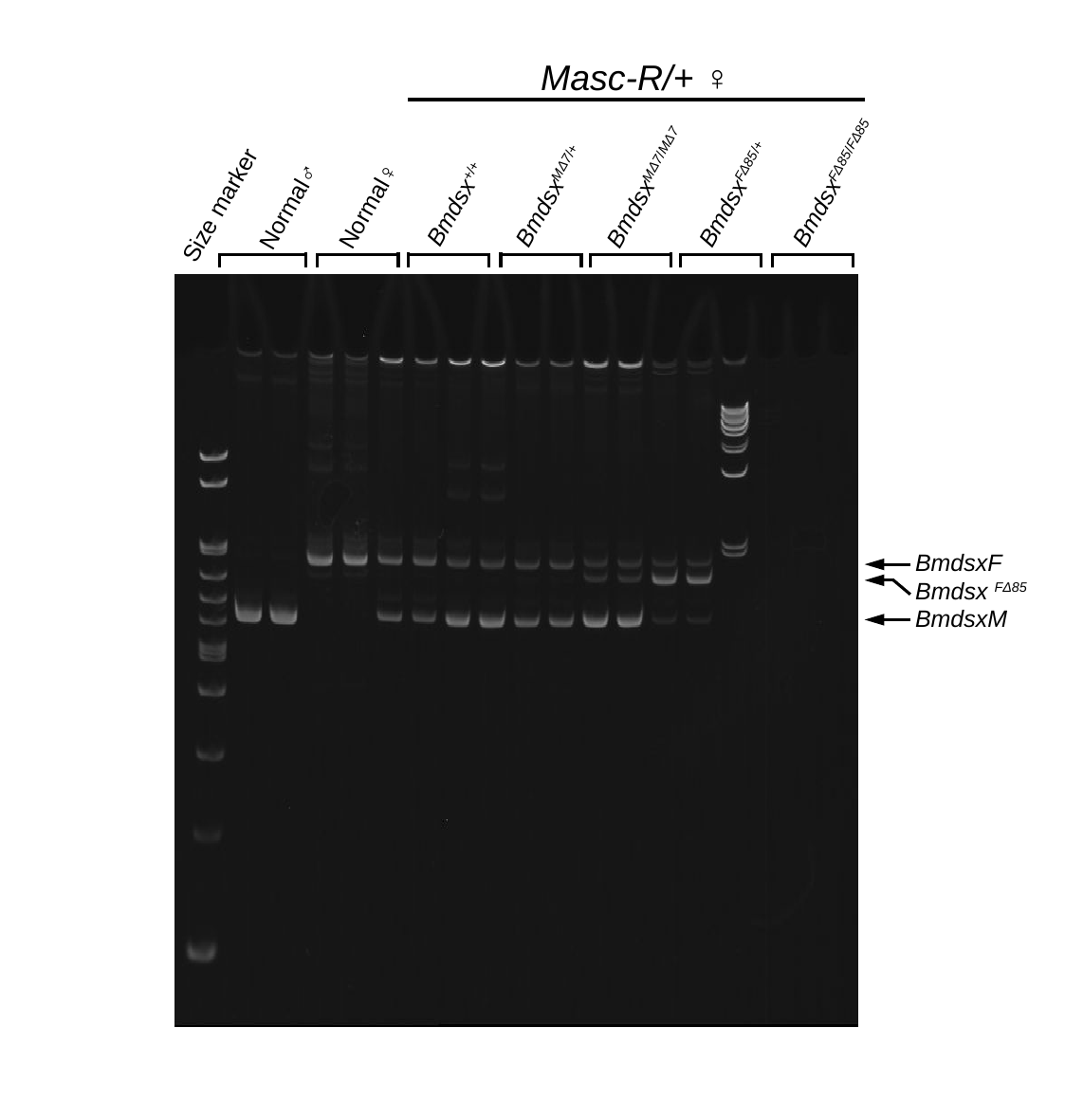

Masc-R/+ ♀
BmdsxFΔ85/FΔ85
BmdsxFΔ85/+
BmdsxMΔ7/MΔ7
BmdsxMΔ7/+
Normal♂
Normal♀
Bmdsx+/+
Size marker
BmdsxF
Bmdsx FΔ85
BmdsxM
