# Supplementary material for: Transgenic and knockout analyses of Masculinizer and doublesex illuminated the unique functions of doublesex in germ cell sexual development of the silkworm, Bombyx mori
Source: BMC Dev Biol. 2020 Sep 21;20:19. doi: 10.1186/s12861-020-00224-2 (PMC7504827; doi:10.1186/s12861-020-00224-2)
Supplement: Supplementary file 9 — Additional file 9: Fig. S5. BmDSX protein levels in BmdsxFΔ85 and BmdsxMΔ7 lines. BmDSX protein levels were determined by western blotting using an anti-DSX-DBD antibody (left panel). Whole protein extracts from testes or ovaries of day-3 5th instar larvae with the indicated genotype were separated by 12.5% SDS-PAGE. The sizes of the molecular markers are indicated on the left. The arrow indicates the protein band corresponding to the molecular weight of each BmDSX protein. The expected molecular weights were as follows: BmDSXM, 32 kDa; BmDSXF, 29.5 kDa; BmDSXMΔ7, 26.6 kDa; BmDSXFΔ85, 24.8 kDa. Histone H3 protein levels were used as loading control (right panel). [file 12861_2020_224_MOESM9_ESM.pptx]

## Slide 1
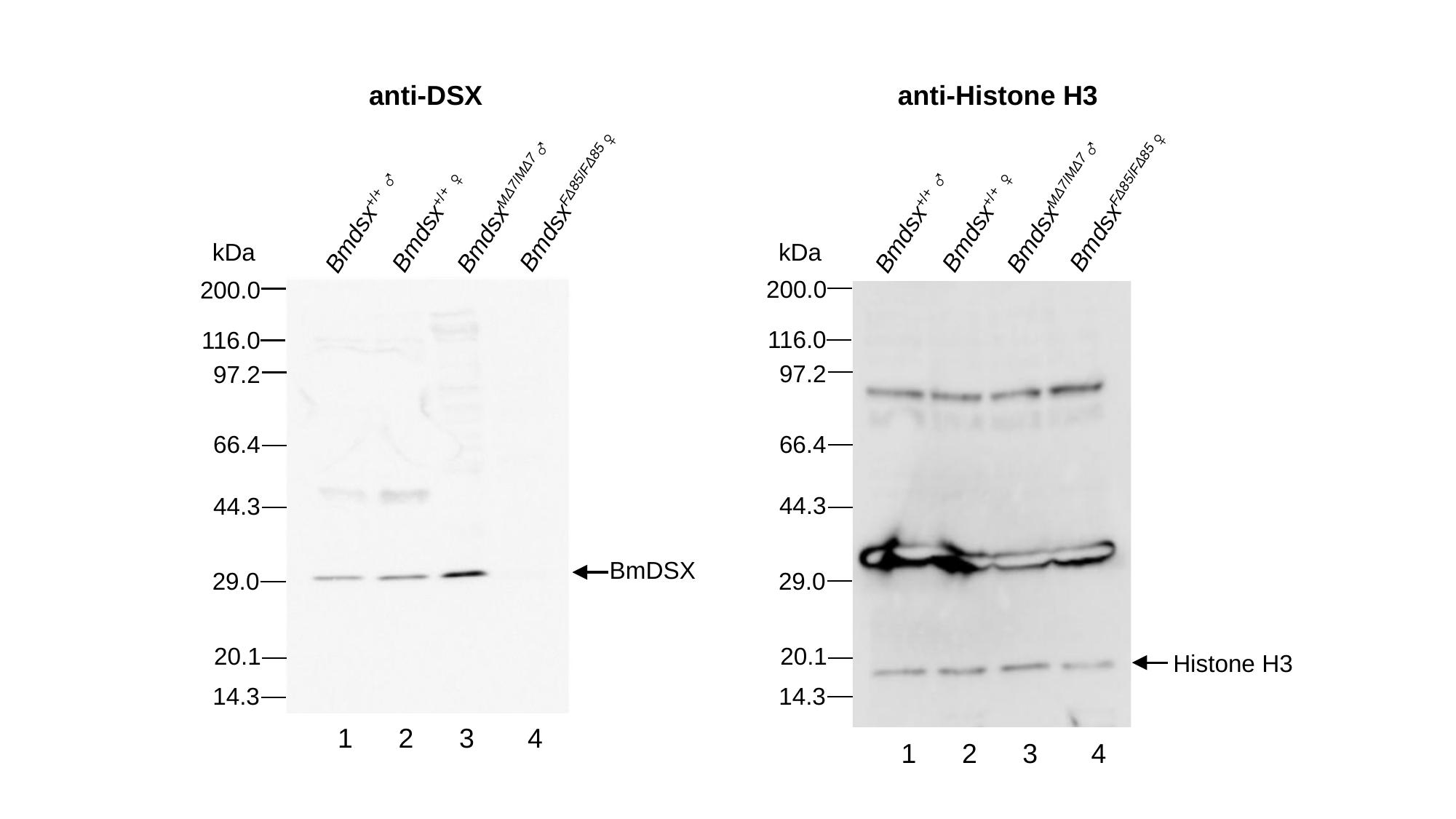

anti-DSX
anti-Histone H3
BmdsxFΔ85/FΔ85♀
BmdsxFΔ85/FΔ85♀
BmdsxMΔ7/MΔ7♂
BmdsxMΔ7/MΔ7♂
Bmdsx+/+ ♀
Bmdsx+/+ ♀
Bmdsx+/+ ♂
Bmdsx+/+ ♂
kDa
kDa
200.0
200.0
116.0
116.0
97.2
97.2
66.4
66.4
44.3
44.3
BmDSX
29.0
29.0
20.1
20.1
Histone H3
14.3
14.3
1 2 3 4
1 2 3 4
