# Supplementary material for: Transgenic and knockout analyses of Masculinizer and doublesex illuminated the unique functions of doublesex in germ cell sexual development of the silkworm, Bombyx mori
Source: BMC Dev Biol. 2020 Sep 21;20:19. doi: 10.1186/s12861-020-00224-2 (PMC7504827; doi:10.1186/s12861-020-00224-2)
Supplement: Supplementary file 10 — Additional file 10: Fig. S6. Procedure followed to generate Masc-R/+ females homozygous for the BmdsxFΔ85 mutation. Masc-R/+ females homozygous for BmdsxFΔ85 were generated by crossing Masc-R and BmdsxFΔ85 animals. (A) In generation 0 (G0), Masc-R/+ females were crossed with males homozygous for the BmdsxFΔ85 mutation. In the next generation (G1), animals heterozygous for the BmdsxFΔ85 mutation were selected after PCR-based genotyping, and females without the Masc-R transgene were crossed with Masc-R/+ males. Individuals carrying the Masc-R transgene were selected based on the expression of the egfp marker gene, as described previously [9]. In the resulting offspring (G2), Masc-R/+ females homozygous for the BmdsxFΔ85 mutation were subjected to further analyses. Individuals heterozygous for the BmdsxFΔ85 mutation or individuals with wild-type BmdsxF were used as controls. (B) PCR-based genotyping for the identification of individuals homozygous or heterozygous for the BmdsxFΔ85 mutation. Genomic PCR was performed as described in Materials and Methods, and the amplified product was separated by 2% agarose gel electrophoresis. The gels were stained with 1% ethidium bromide in 1× TAE buffer to visualize the DNA. The upper bands represent amplicons from wild-type Bmdsx, while the lower bands represent amplicons derived from BmdsxFΔ85 animals. [file 12861_2020_224_MOESM10_ESM.pptx]

## Slide 1
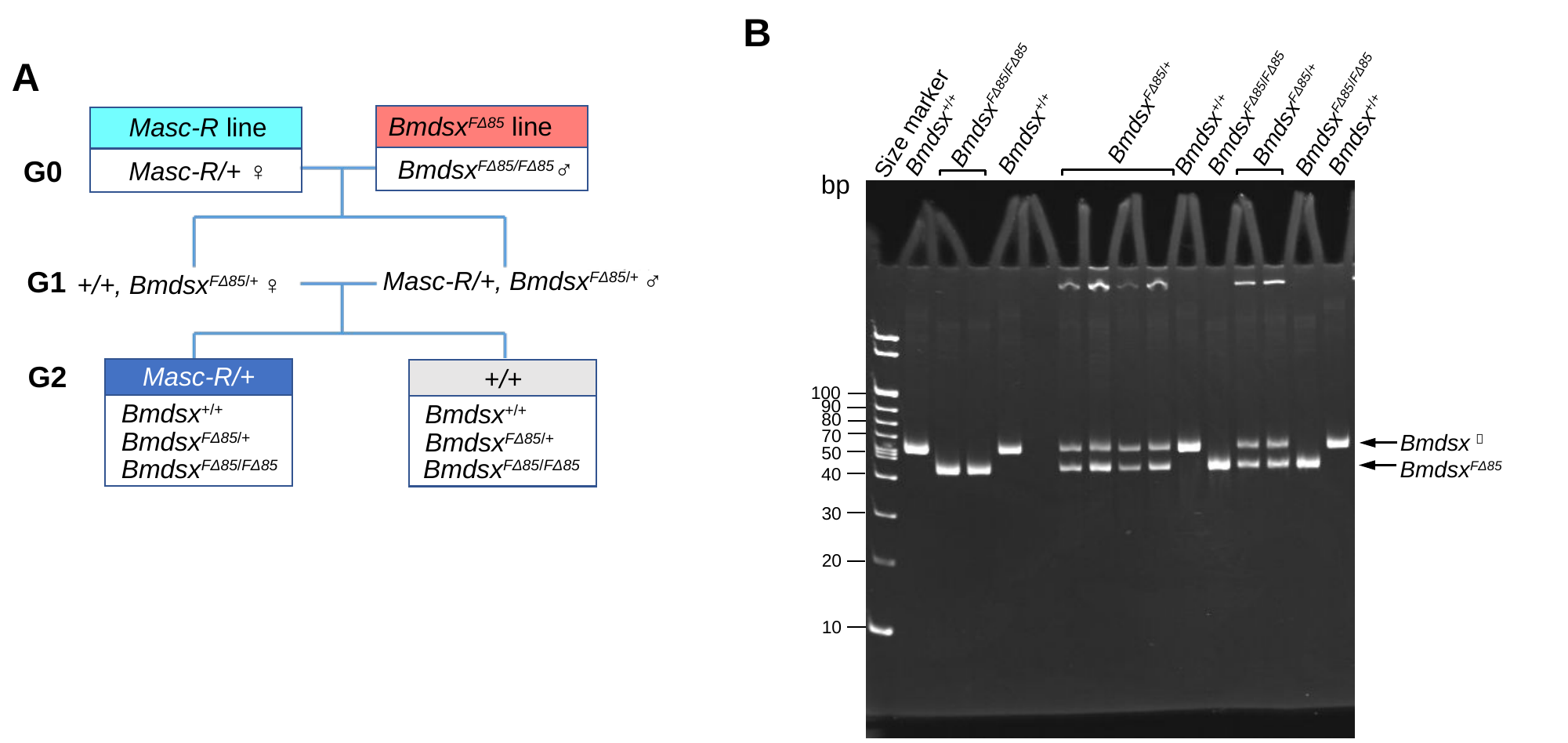

B
A
BmdsxFΔ85/FΔ85
BmdsxFΔ85/FΔ85
BmdsxFΔ85/FΔ85
BmdsxFΔ85/+
BmdsxFΔ85/+
Bmdsx+/+
Bmdsx+/+
Bmdsx+/+
Bmdsx+/+
Size marker
BmdsxFΔ85 line
Masc-R line
Masc-R/+ ♀
G0
BmdsxFΔ85/FΔ85♂
bp
G1
Masc-R/+, BmdsxFΔ85/+ ♂
+/+, BmdsxFΔ85/+ ♀
G2
Masc-R/+
+/+
Bmdsx+/+
BmdsxFΔ85/+
BmdsxFΔ85/FΔ85
100
90
Bmdsx+/+
80
70
BmdsxFΔ85/+
Bmdsx＋
50
BmdsxFΔ85/FΔ85
BmdsxFΔ85
40
30
20
10
