# Supplementary figures and images for: Transgenic and knockout analyses of Masculinizer and doublesex illuminated the unique functions of doublesex in germ cell sexual development of the silkworm, Bombyx mori
Source: BMC Dev Biol. 2020 Sep 21;20:19. doi: 10.1186/s12861-020-00224-2 (PMC7504827; doi:10.1186/s12861-020-00224-2)

## Slide 1
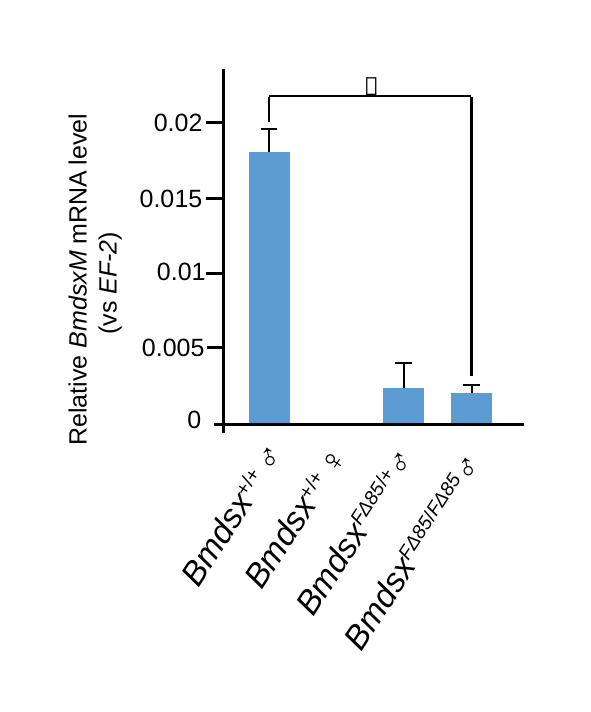

＊
0.02
0.015
 Relative BmdsxM mRNA level
(vs EF-2)
0.01
0.005
0
Bmdsx+/+ ♂
Bmdsx+/+ ♀
BmdsxFΔ85/+♂
BmdsxFΔ85/FΔ85♂

Supplement: Supplementary file 11 — Additional file 11: Fig. S7. Expression levels of BmdsxM in males homozygous for the BmdsxFΔ85 mutation. Expression levels of BmdsxM mRNA in males homozygous for the BmdsxFΔ85 mutation were determined by qRT-PCR. Error bars represent standard deviation. * indicates a significant difference, as determined by Welch’s t-test. [file 12861_2020_224_MOESM11_ESM.pptx]

## Slide 1
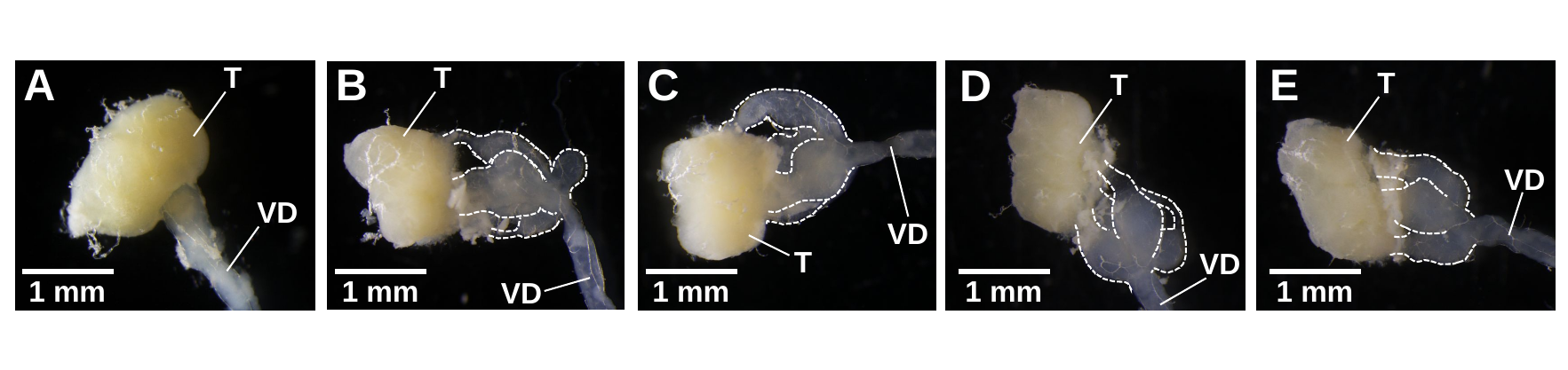

A
E
C
B
D
T
T
T
T
VD
VD
VD
T
VD
1 mm
1 mm
1 mm
1 mm
1 mm
VD

Supplement: Supplementary file 12 — Additional file 12: Fig. S8. Malformed tubes observed at the apical end of the vas deferens in Masc-R females homozygous for the BmdsxF mutation. Images around the apical end of the vas deferens were acquired by a digital camera attached to a stereomicroscope. (A) Normal male. (B–E) Masc-R females homozygous for the Bmdsx FΔ85 mutation. The dotted lines indicate malformed tubes. T: testis, VD: vas deferens. [file 12861_2020_224_MOESM12_ESM.pptx]
